# Metabolic effects of metyrapone treatment in patients with mild autonomous cortisol secretion: a prospective proof-of-concept trial

**DOI:** 10.1016/j.eclinm.2026.103775

**Published:** 2026-02-06

**Authors:** Helena Niziolek, Ivica Just, Anna Tosin, Clemens Baumgartner, Konrad Körmöczi, Luise Bellach, Paul Fellinger, Hannes Beiglböck, Hana Skuciova, Greta Gericke, Stefan Lässer, Anton Luger, Siegfried Trattnig, Alexandra Kautzky-Willer, Marie Helene Schernthaner-Reiter, Florian Wolfgang Kiefer, Greisa Vila, Thomas Scherer, Michael Leutner, Martin Krssak, Michael Krebs, Peter Wolf

**Affiliations:** a Division of Endocrinology and Metabolism, Department of Medicine III, Medical University of Vienna, Vienna, Austria; b Faculty of Mathematics, Physics and Informatics, Comenius University in Bratislava, Bratislava, Slovakia; c Division of Endocrinology and Nephrology, Department of Medicine I, Klinik Landstraße, Vienna, Austria; d Department of Biomedical Imaging and Image Guided Therapy, Medical University of Vienna, Vienna, Austria

**Keywords:** Mild autonomous cortisol secretion (MACS), Adrenal incidentaloma, Chronotherapy, Hypercortisolism, Metyrapone

## Abstract

**Background:**

Mild autonomous cortisol secretion (MACS) is associated with an increased morbidity and mortality. Treatment options range from adrenalectomy to conservative management of comorbidities, but evidence on the effects of medical treatment is scarce. We therefore aimed to investigate the metabolic effects of evening metyrapone treatment in patients with MACS.

**Methods:**

We did a prospective, open-label, proof-of-concept trial (EudraCT: 2022-000161-40). Patients with uni-or bilateral adrenal incidentaloma and MACS defined by cortisol >1·8 μg/dL after 1 mg-dexamethasone-suppression-testing without clinical signs of Cushing's syndrome were included. Participants were investigated at baseline and after 12 weeks of treatment with metyrapone (500 mg at 6 p.m. and 250 mg at 10 p.m.). Intrahepatic lipid content (IHL) and abdominal visceral/subcutaneous fat mass were measured by magnetic resonance spectroscopy and imaging. Resting blood pressure measurements and blood sampling before and during an oral glucose tolerance test were conducted. IHL was the primary outcome parameter. Wilcoxon-signed-rank-tests were used for statistical analysis.

**Findings:**

Between May 2023 and September 2024, 19 patients were enrolled. Fifteen patients were included in the final analysis (12 female, median age 59 years [IQR 53–64]; median BMI 28 kg/m^2^ [25–32]; median cortisol after 1 mg-dexamethasone-suppression-testing 2·9 μg/dL [2·4–4·6]). Metyrapone treatment significantly lowered median IHL at follow up compared with baseline (3·85% of water signal [IQR 1·52–6·58] *vs* 1·92% [1·12–5·91]; p = 0·010). Median fasting insulin (12·6 μlU/mL [IQR 10·5–19·5] *vs* 9·3 μlU/mL [7·2–14·4]; p = 0·041), median c-peptide concentrations (3·0 ng/mL [2·5–4·4] *vs* 2·8 ng/mL [2·2–3·4], p = 0·024) and inflammatory parameters (median leukocyte count 8·1 G/L [6·4–8·9] *vs* 7·4 G/L [6·0–8·8]; p = 0·018; median neutrophil-to-lymphocyte-ratio 2·39 [1·74–2·75] *vs* 2·04 [1·47–2·55]; p = 0·00020) improved. Median systolic (128 mmHg [IQR 122–139] *vs* 122 mmHg [119–126]; p = 0·075) and diastolic (83 mmHg [80–95] *vs* 78 mmHg [75–91]; p = 0·10) blood pressure was non-significantly lower at follow up. No patient reported adverse symptoms of adrenal insufficiency during the study period.

**Interpretation:**

Treatment of MACS with evening doses of metyrapone lowers hepatic lipid content and improves the metabolic risk profile and might offer a novel therapeutic approach.

**Funding:**

10.13039/100017521Esteve (formerly HRA pharma) to the Medical University of Vienna (PI:PW).

**Research in context**

**Evidence before this study**

A PubMed search was conducted in December 2025 using the search terms “autonomous cortisol secretion” OR “adrenal incidentaloma” OR “adrenal mass” OR “subclinical Cushing's syndrome” AND “intervention” OR “medical treatment” OR “metyrapone”. In one identified clinical trial from 2017 Debono et al. reported a restoration of circadian cortisol rhythmicity in patients with mild autonomous cortisol secretion (MACS) after a single day application of evening doses of metyrapone (11β-hydroxylase inhibitor; total daily dose of 750 mg metyrapone). A recent retrospective study by Berry et al. reported improvements in blood pressure by evening metyrapone treatment in patients with MACS. Additionally, Musolino et al. demonstrated beneficial effects of evening metyrapone treatment on blood pressure in patients with mild hypercortisolism. However, data on prospective clinically relevant metabolic outcomes of metyrapone treatment in patients with MACS are limited.

**Added value of this study**

This is the first prospective study examining the metabolic effects of daily evening metyrapone application (500 mg at 6 p.m. and 250 mg at 10 p.m.) for a 12-week treatment period in patients with MACS. Our trial demonstrated a substantial decline of hepatic lipid content, together with improvements in insulin sensitivity, blood pressure and systemic inflammation, indicating a fast re-adaptation of the metabolic risk profile after treatment of hypercortisolism.

**Implications of all the available evidence**

Our results implicate that MACS is associated with an adverse metabolic risk profile and show that this might be improved by treatment with evening doses of metyrapone. This medical treatment strategy might offer a causal therapeutic approach of MACS for patients not eligible for surgery, but might also help to identify patients, who are most likely to benefit from subsequent surgical intervention to guide individualized treatment decisions. Future placebo-controlled studies of longer duration should confirm the observed positive effects of medical treatment.

## Introduction

Adrenal incidentalomas are common in the general population with an estimated prevalence of 1–7%. While most of these tumors can be classified as benign hormonally inactive adenomas without the need for further follow up, about 30% of patients can be diagnosed with mild autonomous cortisol secretion (MACS). MACS therefore is the most common hormonal abnormality in these patients.^1^

Recent clinical practice guidelines define MACS by a morning serum cortisol concentration >1·8 μg/dL after the 1 mg dexamethasone suppression test (DST) in patients with an adrenal adenoma without classical clinical signs of Cushing's syndrome.^2^ Profound evidence suggests that MACS is associated with an increased morbidity and mortality. The prevalence of diabetes, hypertension and dyslipidemia is higher in MACS^3^ and the risk for cardiovascular events is increased.^4^^,^^5^ This adverse metabolic profile results in an increased mortality, particularly in women younger than 65 years of age.^6^

Despite the high prevalence of MACS and its potential clinical impact, therapeutic management of patients is not well defined. Treatment options range from adrenalectomy in patients with a unilateral adrenal mass, to the conservative management of comorbidities,^2^ but evidence on beneficial effects of cortisol-lowering treatment for MACS patients is scarce. Pharmacological treatment options for MACS are of special relevance, as not all patients are candidates for surgery, since they more often have bilateral adenomas or bilateral adrenal hyperplasia.^1^ Moreover, the incidence of MACS demonstrates an age-dependent increase,^7^ rendering elderly patients, particularly those with multiple comorbidities or clinical frailty, less likely to receive treatment in cases where surgical intervention is contraindicated. Short-term drug treatment could also be a useful tool for identifying patients who are most likely to benefit from subsequent surgical intervention to guide individual treatment decisions.

Chronotherapy might be a promising approach for the medical treatment of MACS with the aim to normalize circadian cortisol rhythmicity. In a previous study Debono and colleagues^8^ showed that short-acting cortisol synthesis blockade with evening doses of metyrapone, a selective inhibitor of 11β-hydroxylase, is able to restore the physiological circadian cortisol rhythm by lowering nocturnal cortisol levels without the risk of adrenal insufficiency by overtreatment due to the short half-life of the drug.

The aim of the present trial was to investigate the short-term metabolic effects of medical treatment with evening doses of metyrapone in patients with MACS.

## Methods

### Study design and participants

This prospective, exploratory, open-label, single-center trial (AdrenalClock) was conducted at the Department of Medicine III, Division of Endocrinology and Metabolism, Medical University of Vienna. Patients were recruited between May 2023 and September 2024. They were consecutively enrolled during their regular visits at the outpatient clinic or were recruited by phone calls screened from the adrenal adenoma registry database of the Division of Endocrinology and Metabolism starting with the most recent cases.

Adult patients with uni-or bilateral adrenal adenoma(s) confirmed by computed tomography (CT) scan or magnetic resonance imaging (MRI) and the diagnosis of MACS were included. MACS was defined by a morning cortisol level >1·8 μg/dL without overt clinical signs of Cushing's syndrome.^2^ Exclusion criteria were a HbA1c > 8% and/or insulin therapy, uncontrolled arterial hypertension (requiring daily treatment with >4 antihypertensive drugs), previous glucocorticoid use within the last three months prior to study inclusion, ongoing treatment with drugs strongly affecting *CYP3A4* metabolism, radiological signs suspicious for malignancy, chronic kidney disease (eGFR CKD-EPI<45 mL/min/1·73m2), liver disease (ASAT/ALAT >3 × ULN), pregnancy or breastfeeding. General contraindications for MRI measurements, such as claustrophobia, metal implants, or any other magnetic materials in the body, were considered as additional exclusion criteria.

### Ethics

The trial was conducted in accordance with Good Clinical Practice (GCP) guidelines and the principles of the Declaration of Helsinki. It was approved by the local Ethics Committee of the Medical University of Vienna (1062/2022) and registered at EudraCT (2022-000161-40). Informed consent was obtained from all participants.

### Procedures

Patients were investigated at baseline and after 12 weeks of treatment with evening doses of metyrapone (Metycor ®) with a dose of 500 mg at 6 p.m. and 250 mg at 10 p.m., according to the protocol by Debono et al.^8^ No metyrapone dosage adaptions were performed during the trial period. Participants were carefully informed about potential adverse effects and symptoms of adrenal insufficiency. They were instructed to withhold the study medication on sick days and were provided with appropriate rescue medication with hydrocortisone in case of severe illness during the period of the clinical trial.

The following examinations were systematically performed in all patients before and after treatment:

Anthropometric characteristics such as height, weight, body mass index and waist circumference were assessed. Information on medical history and concomitant medication was received by self-report of the patients and extractions from clinical records. Adrenal mass diameter was reported. In case of bilateral disease, the diameter of the larger lesion is given. Blood pressure was measured in supine position following at least 5 min of rest. The presence of hypertension was defined by medical history of hypertension, a blood pressure of >140/90 mmHg at the study visit or the concomitant intake of anti-hypertensive medication. The presence of type 2 diabetes mellitus was defined by medical history of type 2 diabetes mellitus, an HbA1c ≥ 6·5% at the study visit or the concomitant intake of anti-diabetic medication.

Body composition (fat mass and fat free mass) was assessed using the BOD POD system (BOD POD GS-X, Cosmed, Rome, Italy), an air displacement plethysmography device.^9^

Blood was drawn in the morning after an overnight fast to measure concentrations of hormones (serum cortisol, adrenocorticotropic hormone [ACTH], dehydroepiandrosterone sulfate [DHEAS], 17-hydroxyprogesterone [17-OH-progesterone], 11β-deoxycortisol [11-DOC], androstenedione, testosterone), electrolytes, inflammatory parameters (platelets, leukocytes, neutrophils, lymphocytes, monocytes and c-reactive protein), parameters of glucose and lipid metabolism (HbA1c, glucose, insulin, c-peptide, triglycerides, total cholesterol, HDL-cholesterol, LDL-cholesterol) and parameters of kidney and liver function. An oral glucose tolerance test (OGTT) was performed with additional venous blood samples at 30, 60, 90, and 120 min following the ingestion of 75 *g* of glucose for the measurement of glucose, insulin, and c-peptide concentrations. Estimates of hepatic (HOMA-IR) and peripheral (OGIS) insulin resistance were calculated as previously reported.^10^ Participants were furthermore instructed to collect late-night salivary cortisol (LNSC) at 12:00 p.m. (two consecutive saliva samples on the same evening, mean values were used for analysis) and to collect urine for 24 h for the assessment of urinary free cortisol (UFC) excretion the day before both study days. All laboratory parameters were measured at the routine laboratory of the Medical University of Vienna by immunoassays using standard analytical methods (https://labormedizin.meduniwien.ac.at/).

All patients underwent ^1^H-magnetic resonance spectroscopy (^1^H-MRS) and imaging using a high-field 3T whole body MR system (Magnetom, Prisma Fit, Siemens Healthineers, Erlangen, Germany). Intracellular hepatic lipid (IHL) content was determined using single voxel ^1^H-MRS, based on methylene and methyl resonance signals, which were corrected for longitudinal and individual transversal relaxation times and expressed as a percentage of the total MRS signal (including water, methylene and methyl peaks), in accordance with previously published methods.^11^^,^^12^ Abdominal visceral and subcutaneous fat was quantified from T_1_-weighted images in three cross-sectional places in the abdomen–at the level of the intervertebral disc between L4/L5 vertebrae, L3/L4 and L2/L3 using automatic segmentation based on 2D Unet architecture.

The relative change in hepatic lipid content was calculated with the formula: ((liver fat fraction at baseline—liver fat fraction on Day 2)/baseline liver fat fraction) × 100.

For safety monitoring and to assure compliance with the study drug, patients were additionally investigated clinically after four- and eight-weeks following trial initiation. At these study visit, patients were systematically asked about adverse events and serious adverse events and were supplied with the study medication for the following 4 weeks.

### Outcomes

The primary outcome parameter was intrahepatic lipid content, defined as a percentage of the total water MRS signal (% of w.s.). The primary endpoint was the change of intrahepatic lipid content at follow up compared to baseline.

Exploratory secondary outcome parameters were systolic and diastolic blood pressure, measured in supine position after at least 5 min of rest (mmHg), body composition, defined as total mass, fat mass and fat free mass (kg), visceral and subcutaneous abdominal adipose tissue (mL), parameters of glucose metabolism (fasting glucose (mg/dL), insulin (μlU/mL), c-peptide (ng/mL), HbA1c (%), HOMA-IR, OGIS (ml·min^−1^·m^−2^)) and lipid metabolism (total cholesterol (mg/dL), HDL- and LDL-cholesterol (mg/dL) and triglycerides (mg/dL)), inflammatory parameters (leucocytes (G/L), neutrophiles (G/L, lymphocytes (G/L), CRP (mg/dL), ratios of neutrophiles-to-lymphocytes (%) and lymphocytes-to-leukocytes (%)) and surrogates of hypothalamus-pituitary-adrenal axis activity (ACTH (pg/mL), cortisol (μg/dL), 24 h UFC (μg/24 h), LNSC (μg/dL), DHEAS (μg/mL), 11-Deoxycortisol, ng/mL) assessed at baseline and follow up.

### Statistics

Based on previous studies on short term treatment effects on hepatic lipid content (primary outcome parameter) in other endocrine diseases^13^^,^^14^ we expected to observe a 25% reduction at follow up. A sample size of 15 patients was determined in power calculations to observe intra-individual changes with alpha 0·05 and beta 0·8. Drop-outs were not included in the sample size and were replaced by allocation of the next free subject number according to the study protocol. No blinding or randomization was conducted due to the exploratory proof-of concept setting. For the secondary endpoints (insulin secretion and sensitivity based-indices, subcutaneous and visceral fat mass, body composition, blood pressure, parameters of inflammation) no separate sample size calculation was performed.

Continuous variables were reported as medians (Q1–Q3), categorical variables were presented as absolute numbers and corresponding percentages. Area under the curve (AUC) was calculated using the trapezoidal rule. Given the small sample size and variable distribution, a non-parametric approach was chosen and Wilcoxon signed-rank tests applied to compare intra-individual differences at baseline vs. follow-up. Associations between quantitative outcomes and the primary outcome parameter (hepatic lipid content) were analyzed with the Spearman's rank correlation coefficient. A robust linear regression model was applied due to the small sample size and presence of outliers, which were visually determined based on regression diagnostic plots. Two-sided p-values were calculated using the formula: 2∗(1-pt (abs (t-value), number of degrees of freedom)). Statistical analysis was performed using R (Version 2023.12.1 + 402) and GraphPad Prism Version 10 (GraphPad Software, Boston, Massachusetts, USA). P-values less than 0·05 were considered statistically significant.

### Role of the funding source

The trial was in parts financially supported by an investigator-initiated grant from *ESTEVE* (formerly HRA pharma) to the Medical University of Vienna (PI: PW). Funding sources did not affect or modify trial design, outcome measures and data analysis.

## Results

**Fig. 1:**
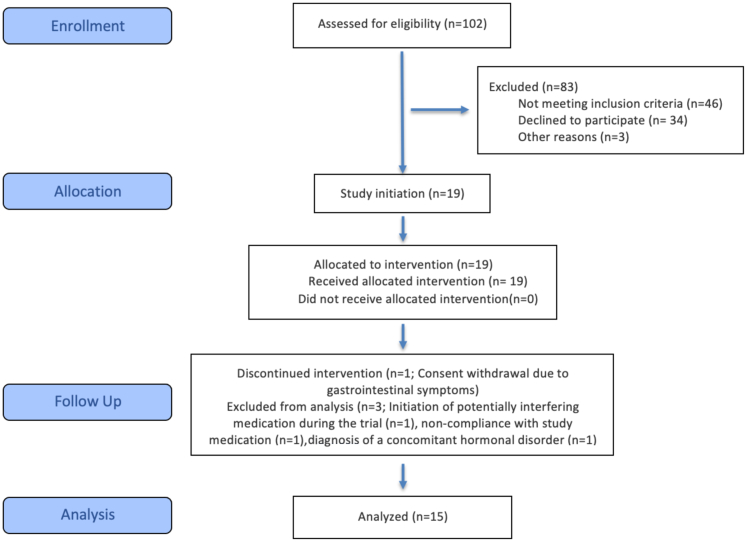
Flow diagram of the progress through the phases of the proof-of-concept trial (that is, enrollment, intervention, follow-up, and data analysis).

19 patients were enrolled between May 2023 and September 2024. One participant withdrew consent during the study period due to gastrointestinal symptoms probably attributed to metyrapone. Three patients were excluded for the following reasons: initiation of GLP-1 receptor agonist therapy during the study period by her general practitioner, non-compliance with study medication and diagnosis of a concomitant hormonal disorder known to affect metabolism during the clinical trial. 15 patients were included in the final analysis and completed all measurements at baseline and follow-up (Fig. 1).

Baseline anthropometric, disease-related clinical characteristics of the study cohort are summarized in Table 1. 12 (80%) participants were women. Seven (47%) patients had bilateral adenomas. All patients had suppressed or low ACTH concentrations (≤10 pg/ml) at baseline.

**Table 1:** Baseline characteristics reported as median (Q1–Q3) or absolute number (%).

Baseline characteristics	
Number of patients	15
Age in years (Q1–Q3)	59 (53–64)
Sex (females in %)	12 (80)
Unilateral disease, n (%)	8 (53)
Tumor diameter in mm (Q1–Q3)	25·0 (19·5–34·5)
Body weight in kg (Q1–Q3)	75·2 (69·8–85.0)
Height in cm (Q1–Q3)	162 (158–169)
BMI in kg/m^2^ (Q1–Q3)	28 (25–32)
Waist circumference in cm (Q1–Q3)	100 (93–107)
Cortisol after DST in μg/dl (Q1–Q3)	2·9 (2·4–4·6)
Presence of arterial hypertension, n (%)	12 (80)
Presence of diabetes mellitus type 2, n (%)	3 (20)

In case of bilateral lesions, the larger tumor diameter is reported; Abbreviations: BMI, body mass index; DST, dexamethasone suppression test.

**Fig. 2:**
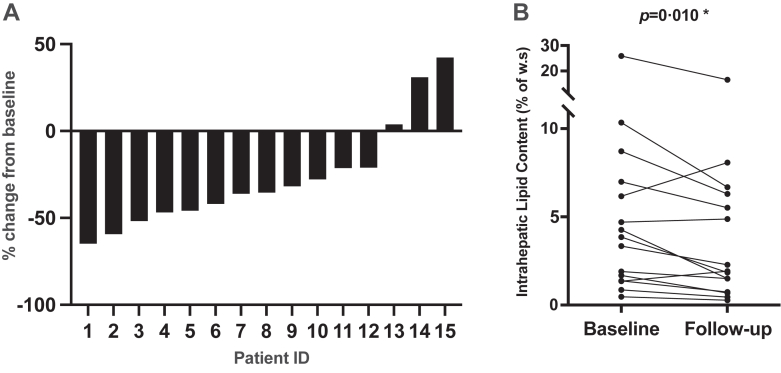
(A) Percentage change of hepatic lipid content per patient after 12 weeks of metyrapone treatment, (B) comparison of hepatic lipid content (% of w.s) at baseline and after 12 weeks of treatment. Wilcoxon signed-rank test was applied for comparison of hepatic lipid content at baseline and follow-up. Abbreviations: w.s, water signal. ∗Indicates statistical significance p < 0·05.

Following 12 weeks of metyrapone treatment, we observed a significant reduction in median hepatic lipid content (3·85% of water signal [IQR 1·52–6·58] *vs* 1·92% [1·12–5·91]; p = 0·010; Fig. 2). Abdominal visceral and subcutaneous adipose tissue, body composition, waist circumference and total body weight did not change (Supplemental Figure S1).

**Fig. 3:**
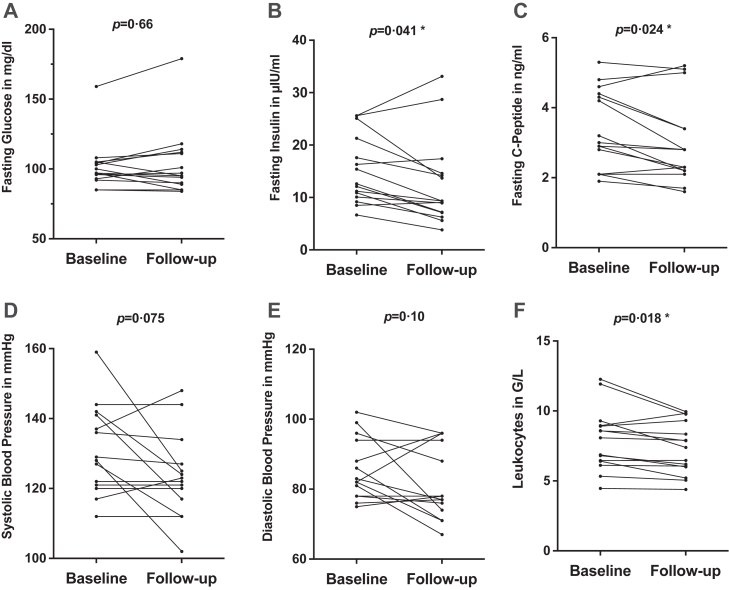
Comparison of fasting glucose (A), fasting insulin (B), fasting c-peptide (C), systolic blood pressure (D), diastolic blood pressure (E), and leukocytes (F) at baseline and after 12 weeks of metyrapone treatment with Wilcoxon signed-rank tests. ∗Indicates statistical signficance p < 0·05.

Fasting glucose concentrations did not change with treatment, but median fasting insulin (12·6 μlU/mL [IQR 10·5–19·5] *vs* 9·3 μlU/mL [7·2–14·4]; p = 0·041) and c-peptide levels (3·0 ng/mL [2·5–4·4] *vs* 2·8 ng/mL [2·2–3·4], p = 0·024) were significantly lower at follow up compared with baseline (Fig. 3; A, B, C). In line with this improvement in insulin sensitivity, non-significant improvements in HOMA-IR and OGIS as surrogate parameters for hepatic and peripheral insulin resistance were observed (Supplemental Figure S2). Evaluation of lipid parameters displayed no changes in concentrations of total cholesterol, triglycerides and LDL-cholesterol, whereas HDL-cholesterol decreased after treatment.

Median systolic (128 mmHg [IQR 122–139] *vs* 122 mmHg [119–126]; p = 0·075) and diastolic (83 mmHg [80–95] *vs* 78 mmHg [75–91]; p = 0·10) blood pressure declined, but non-significantly (Fig. 3; D, E). 12 of 15 patients took antihypertensive medication at baseline. Four of these 12 patients independently reduced or discontinued their antihypertensive medication due to lower home blood pressure self-measurements, which may have attenuated the observed treatment effects. Detailed information on concomitant treatment with antihypertensive medication at baseline and follow up is reported in the Supplemental Table S1.

With regard to inflammation parameters, the median absolute counts of leucocytes (7·4 G/L [IQR 6·0–8·8] *vs* 8·1 G/L [6·4–8·9]; p = 0·018; Fig. 3 F) and neutrophils (4·3 G/L [3·17–4·68] *vs* 4·75 G/L [4·05–6·05]; p = 0·0042) were significantly lower at follow-up compared with baseline. This resulted in a significant reduction in the median ratio of neutrophils-to-lymphocytes (2·04% [1·47–2·55] *vs* 2·39% [1·74–2·75]; p < 0·001) and ratio of lymphocytes-to-leukocytes (3·37% [2·76–3·96] *vs* 3·69% [3·08–4·08]; p < 0·001). No differences in CRP levels were observed (0·2 mg/dL [0·0–0·3] *vs* 0·1 mg/dL [0·1–0·3]; p = 0·31). Data are reported in detail in Table 2.

**Table 2:** Descriptive statistics of all outcomes (median, Q1–Q3) before and after 12 weeks of treatment with metyrapone.

	Before treatment (n = 15)	After treatment (n = 15)	p-value
Clinical and biochemical parameters			
Waist Circumference, cm	100·0 (93·0–106·5)	101·5 (91·5–103·5)	0·78
Weight, kg	75·2 (69·8–85·0)	75·4 (69·1–84·7)	0·49
BMI	28·3 (25·3–31·9)	27·7 (25·1–31·7)	0·36
Body fat, %	45·1 (34·4–51·0)	45·2 (34·3–52·1)	0·57
Total body fat mass, kg	32·2 (25·8–42·5)	29·9 (25·7–44·4)	0·39
Total body fat free mass, kg	42·1 (38·1–45·2)	42·5 (38·1–45·7)	0·93
Potassium, mmol/L	4·49 (4.39–4.6)	4·35 (4.21–4.47)	0·37
Systolic blood pressure, mmHg	128 (122–139)	122 (119–126)	0·075
Diastolic blood pressure, mmHg	83 (80–95)	78 (75–91)	0·10
MRI & proton spectroscopy (Median, q1–q3)			
Hepatic lipid content, % of w.s. (n = 15)	3·85 (1·52–6·58)	1·92 (1·12–5·91)	**0**·**010**
Total visceral fat mass, ml	500·4 (435·7–597·4)	458·7 (388·0–612·5)	0·30
Total subcutaneous fat mass, ml	904·2 (526·4–1102·2)	888·1 (544·8–1011·6)	0·80
Ratio visceral fat mass/subcutaneous fat mass	0·56 (0·47–0·86)	0·57 (0·47–0·84)	**1**
Metabolic parameters			
HbA1c, %	5·6 (5·4–6·0)	5·9 (5·4–6·2)	0·12
HOMA_IR	3·14 (2·51–4·63)	2·16 (1·60–4·20)	0·14
OGIS,ml·min^−1^·m^−2^	370 (327–395·5)	354·5 (309·75–366·25)	0·069
Fasting glucose, mg/dL	97·0 (94·5–104·5)	95·0 (89·5–111·5)	0·66
Fasting insulin, μlU/mL	12·6 (10·5–19·5)	9·3 (7·2–14·4)	**0**·**041**
Fasting C-peptide,ng/mL	3·0 (2·5–4·4)	2·8 (2·2–3·4)	**0**·**024**
AUC_Glucose,_ mg^a^min/dL	19,230 (16,673–22448)	20,685 (17,625–22238)	0·71
AUC_Insulin,_ μlU^a^min/mL	7313 (5409–11870)	8895 (6888–12197)	0·24
AUC_C-Peptide,_ ng^a^min/mL	1187 (892–1377)	1223 (1116–1384)	0·30
Total cholesterol, mg/dL	200 (183–216)	177 (151–215)	0·23
HDL cholesterol, mg/dL	54 (45–67)	47 (44–62)	**0**·**025**
LDL cholesterol, mg/dL	117 (92–133)	84 (72–120)	0·33
Triglycerides, mg/dL	131 (103–191)	144 (116–196)	0·93
Blood count and inflammatory markers			
Platelets, G/L	281 (230–366)	276 (220–361)	0·36
Leukocytes, G/L	8·1 (6·4–8·9)	7·4 (6·0–8·8)	**0**·**018**
Neutrophils, G/L	4·75 (4·05–6·05)	4·3 (3·17–4·68)	**0**·**0042**
Lymphocytes, G/L	2·10 (1·80–2·58)	2·10 (1·90–2·50)	0·48
Monocytes, G/L	0·50 (0·40–0·65)	0·50 (0·40–0·67)	0·90
NLR%	2·39 (1·74–2·75)	2·04 (1·47–2·55)	**0**·**00020**
LLR%	3·69 (3·08–4·08)	3·37 (2·76–3·96)	**0**·**00020**
PLR%	126·35 (110·12–146·15)	130·79 (96·41–180·00)	0·64
MLR%	0·22 (0·17–0·30)	0·23 (0·18–0·28)	0·79
CRP, mg/dL	0·1 (0·1–0·3)	0·2 (0·0–0·3)	0·31
Hormone parameters			
Serum-Cortisol, μg/dL	11·70 (9·98–14·38)	15·10 (13·10–18·50)	0·13
Free Cortisol, ng/mL	5·45 (3·83–6·78)	6·75 (5·30–8·32)	0·40
ACTH, pg/mL	4 (4–6)	7 (4–13)	**0**·**010**
DHEAS, μg/mL	0·46 (0·26–0·83)	0·51 (0·30–0·94)	0·09
Testosterone, ng/mL	0·10 (0·03–0·22)	0·33 (0·18–0·67)	0·29
Bioavailable testosterone, ng/mL	0·04 (0·01–0·06)	0·11 (0·05–0·18)	0·41
Androstenedione, ng/mL	0·47 (0·38–0·68)	1·48 (1·06–1·99)	**0**·**00010**
17-hydroxyprogesterone, ng/mL	0·64 (0·34–1·37)	1·02 (0·69–2·26)	**0**·**020**
11-Deoxycortisol, ng/mL	2·97 (2·17–4·30)	12·52 (7·69–15·22)	**0**·**00010**
Mean^a^ 24 h urinary free cortisol, μg/24 h	74·72 (57·71–123·95)	62·00 (44·86–84·29)	0·46
Mean^a^ late night saliva cortisol, μg/dL	0·07 (0·05–0·08)	0·05 (0·05–0·06)	0·22

All biochemical parameters were measured in the morning after an overnight fast and were compared with Wilcoxon signed-rank tests. Abbreviations: ACTH, adrenocorticotropic hormone; AUC, area under the curve; BMI, body mass index; CRP, C-reactive protein; DHEAS, dehydroepiandrosterone sulfate; HbA1c, glycated hemoglobin; HDL, high density lipoprotein; HOMA_IR, Homeostasis Model Assessment of Insulin Resistance; LDL, low density lipoprotein; LLR, leukocyte-to-lymphocyte ratio; MLR, monocyte-to-lymphocyte ratio; NLR, neutrophil-to-lymphocyte ratio; OGIS, Oral Glucose Insulin Sensitivity Index; PLR, platelet-to-lymphocyte ratio; w.s., water signal.

Significan values (p < 0·05) are indicated in bold.

a Mean values for 24 h urinary free cortisol and midnight saliva cortisol were calculated from two samples per patient. In case of only one available sample the single value was used.

Median morning cortisol levels (15·10 μg/dL [IQR 13·10–18·50] *vs* 11·70 μg/dL [9·98–14·38]; p = 0·13), LNSC (0·05 μg/dL [0·05–0·06] *vs* 0·07 μg/dL [0·05–0·08]; p = 0·22) and 24-h UFC excretion (62·00 μg per 24 h [44·86–84·29] *vs* 74·72 μg per 24 h [57·71–123·95]; p = 0·46) did not change throughout the trial. Median ACTH was significantly higher at follow-up compared with baseline (7 pg/mL [4–13] *vs* 4 pg/mL [4–6]; p = 0·01; Table 2). As indicator of compliance with the treatment, median 11-deoxycortisol (12·52 ng/mL [7·69–15·22] vs 2·97 ng/mL [2·17–4·30]; p < 0·001), androstenedione (1·48 ng/mL [1·06–1·99] *vs* 0·47 ng/mL [0·38–0·68]; p < 0·001) and 17-hydroxyprogesterone (1·02 ng/mL [0·69–2·26] *vs* 0·64 ng/mL [0·34–1·37]; p = 0·020) concentrations were higher at follow-up compared with baseline (Supplemental Figure S3 and Supplementary Table S2).

The one patient who withdrew consent during the study period reported self-limiting symptoms indicative for a gastrointestinal tract infection (fever, gastro-intestinal symptoms, abdominal pain), but a possible relationship with the study drug cannot be excluded. All other patients did not report any clinical signs of adrenal insufficiency. No serious adverse effects were reported by the patients (Table 3). A detailed summary of all outcome parameters is reported in Table 2.

**Table 3:** Reported adverse events during study period.

Adverse events	Number of patients (%)
Diarrhea and abdominal pain	1 (5·2)
Other adverse reactions	0 (0)

Baseline hepatic lipid content showed a positive correlation with waist circumference (r = 0·670, p = 0·0063), BMI (r = 0·621, p = 0·016), total body fat mass (r = 0·650, p = 0·011), percentage of body fat (r = 0·607, p = 0·019), HOMA-IR (r = 0·564, p = 0·031), fasting insulin (r = 0·586, p = 0·022), AUC of insulin during OGTT (p = 0·554, p = 0·035) and a negative correlation with 24 h urinary free cortisol (r = −0·671, p = 0·020).

The decline in hepatic lipid content strongly correlated with fasting glucose (r = −0·525, p = 0·044), c-peptide (r = −0·626, p = 0·013), the percentage of body fat (r = −0·564, p = 0·031) and 24-h UFC (r = 0·741, p = 0·0082) at baseline. A robust linear regression model was applied due to the limited sample size and presence of outliers, including fasting glucose, 24-h UFC, and percentage body fat as covariates. In the robust linear regression model a significant association between baseline fasting glucose (r = −0·560, p-value = 0·0019) and percentage of body fat (estimate = −0·626, p-value = 0·029) with decrease in hepatic lipid content (Supplemental Table S2) was determined.

## Discussion

In this prospective, open-label, proof-of-concept trial we evaluated the metabolic effects of treatment with evening doses of metyrapone in patients with MACS. We observed a significant reduction in intrahepatic lipid content (primary outcome parameter) together with improvements in insulin sensitivity, blood pressure and systemic inflammation after 12 weeks of treatment. The results of our trial indicate that MACS is associated with an adverse metabolic risk profile, which might be improved by pharmacological treatment with evening doses of metyrapone.

Hepatic lipid content was lower in 12 of 15 patients after treatment, resulting in a mean reduction of 27% at follow-up compared to baseline (Fig. 2). Hepatic lipid content was chosen as primary outcome parameter as it is a well-established, sensitive surrogate marker of metabolism.^15^ When assessed by ^1^H-MRS, it offers high reproducibility with low intra-individual day-to-day variability and minimal inter-observer variability.^12^^,^^16^ Furthermore, ectopic fat has a higher plasticity and is more susceptible to short-term interventions compared to conventional white adipose depots.^13^ This might explain the observed changes in hepatic lipid content in a short term follow-up after 12 weeks of treatment, while body composition, visceral and subcutaneous abdominal adipose depots did not change significantly.

An improvement in insulin sensitivity was observed in our trial, indicated by the reduction of fasting insulin and c-peptide concentrations following treatment with metyrapone. In line with our results, improvements in glucose metabolism were shown in patients with MACS after unilateral adrenalectomy.^17^ Evaluation of lipid parameters displayed a decline of HDL-cholesterol after treatment, while total cholesterol, LDL-cholesterol and triglycerides remained unchanged. The underlying mechanistic pathway cannot be explained by our data and should be further investigated in future trials.

Studies investigating the effects of adrenalectomy in patients with MACS report improvements in blood pressure^17^, ^18^, ^19^ A retrospective analysis of patients with MACS treated with evening doses of metyrapone reported a reduction of both systolic and diastolic blood pressure,^20^ while Musolino et al. showed a decline in systolic 24h-blood pressure after evening metyrapone treatment in patients with mild hypercortisolism.^21^ In our study systolic and diastolic blood pressure were non-significantly lower after 12 weeks of treatment with metyrapone. Of note, four out of 12 patients using antihypertensive medication at time of trial inclusion reduced their concomitant antihypertensive medication by themselves throughout the trial period (see Supplementary Table S1), which might have attenuated the statistical significance.

A low-grade inflammatory state was described in patients with hypercortisolism.^22^^,^^23^ Following treatment with metyrapone, we observed a significant decline in absolute concentrations of leucocytes and neutrophiles, resulting in changes in the ratio of neutrophiles-to-lymphocytes and the ratio of lymphocytes-to-leukocytes.

An important finding of our trial is that treatment with evening doses of metyrapone was overall well tolerated and might be a safe approach for the medical treatment of MACS. One patient reported diarrhea and withdraw from the trial, otherwise no clinical signs of adrenal insufficiency were observed throughout the trial period. We found changes in ACTH, 17-hydroxyprogesterone and 11-deoxycortisol, which shows an effect of Metyrapone on cortisol production.

The major limitation of our trial is the missing placebo control group. As this trial is one of the first to investigate metabolic outcomes of metyrapone treatment in MACS, it was designed as a proof-of-concept trial. Efforts to minimize bias were made through objective biochemical assessments, as well as by following pre-existing implemented routine clinical procedures during the study visits. Regarding the study cohort, four patients did not have a second confirmatory dexamethasone suppression test before study inclusion. A threshold value for ACTH was also not defined in the study protocol. However, all study participants had ACTH levels ≤10 pg/ml and low DHEAS concentrations at baseline. In addition, the small sample size leads to risk for type 1 and type 2 errors. Our trial cohort was not well balanced for sex, therefore sex-specific conclusions cannot be drawn. Steroid metabolite cross-reactivity by using immune-assays might also have an impact on our results.

Despite applying a therapeutic approach of chronotherapy, we could not confirm improvements in circadian cortisol rhythmicity in our trial as late night salivary cortisol concentrations were not different before and after treatment. Multiple time-point assessments of salivary specimen could have been more sensitive and should be conducted in future trials. Previous studies show that late night salivary cortisol is not useful in patients with MACS, as it is rapidly converted to cortisone in the salivary gland.^24^^,^^25^ Salivary cortisone might have a better discriminatory potential to compare patients with MACS compared to non-functioning adrenal adenomas. However, we did not assess salivary cortisone concentrations in this trial because of methodological limitations.

In conclusion, our results suggest that MACS is associated with an adverse metabolic risk profile, which is likely improved by medical treatment with evening doses of metyrapone. Metyrapone treatment may represent a novel clinical tool to identify patients who are most likely benefiting from subsequent surgical intervention to individualize treatment decisions but could also represent a therapeutic alternative for patients with MACS not suitable for surgery. The results of our findings should be confirmed in future randomized placebo-controlled trials of longer duration.

## Contributors

All authors read and approved the final version of the manuscript. The data was verified by H.N. and P.W.

Author contributions: Concept of the trial (P.W., M.Kre.), recruitment of patients (P.W., H.N., C.B., H.B., L.B, P.F., G.G., S.L., MH.SR., F.W.K., A.L., A.KW., T.S., G.V., M.L., M.Kre.), data collection and performance of experiments (P.W., H.N., I.J., K.K., H.S., M.Krs., S.T., A.T., C.B., P.W.), statistical analysis (H.N., P.W.), drafting of the manuscript (H.N., P.W.) and revision for important intellectual content as well as approval of the final manuscript (all authors).

## Data sharing statement

The data underlying this article will be shared upon reasonable request to the corresponding author.

## Declaration of interests

PW received honoraria for lectures and presentations from Recordati rare diseases and HRA Pharma, participated in advisory boards organized by Recordati rare diseases and HRA Pharma, and received support for attending meetings from HRA Pharma. GV received advisory and/or speaker fees from Lundbeck, Recordati and HRA-Pharma; holds the position as local PI in multicentric studies from Recordati and Corcept; and was an editor for Cushing's Hub (Elsevier). MHSR received honoraria for lectures from Recordati rare diseases and HRA Pharma, participated in advisory boards organized by Recordati rare diseases and HRA Pharma, and received support for attending meetings from Recordati and HRA Pharma. All other authors do not have a potential conflict of interest to report.

## Appendix A Supplementary data

Supplementary Tables and Figures

Study Protocol
