# Supplementary material for: Metabolic effects of metyrapone treatment in patients with mild autonomous cortisol secretion: a prospective proof-of-concept trial
Source: eClinicalMedicine. 2026 Feb 6;92:103775. doi: 10.1016/j.eclinm.2026.103775 (PMC12907678; doi:10.1016/j.eclinm.2026.103775)
Supplement: Supplementary Tables and Figures [file mmc1.docx]

**Supplementary tables and figures**

**Metabolic effects of metyrapone treatment in patients with mild autonomous cortisol secretion: a prospective proof-of-concept trial**

Helena Niziolek, MD^1^, Ivica Just,PhD^1^, Anna Tosin^1^, Clemens Baumgartner,MD^1^, Konrad Körmöczi^1^, Luise Bellach, MD ^1^, Paul Fellinger, MD ^1^,Hannes Beiglböck, MD PhD^1^, BSc Hana Skuciova^1,2^, Greta Gericke, MD ^3^, Stefan Lässer, MD ^3^, Prof. Anton Luger, MD^1^, Prof. Siegfried Trattnig,MD^4^, Prof. Alexandra Kautzky-Willer,MD^1^, Marie Helene Schernthaner-Reiter, MD PhD^1^, Florian Wolfgang Kiefer, MD PhD^1^, Greisa Vila, MD^1^, Prof. Thomas Scherer,MD PhD^1^, Michael Leutner,MD PhD^1^, Martin Krssak,PhD^1^, Michael Krebs,MD^1^, Peter Wolf, MD PhD^1^

1. Division of Endocrinology and Metabolism, Department of Medicine III, Medical University of Vienna, Vienna, Austria
2. Faculty of Mathematics, Physics and Informatics, Comenius University in Bratislava, Bratislava, Slovakia
3. Division of Endocrinology and Nephrology, Department of Medicine I, Klinik Landstraße, Vienna, Austria
4. Department of Biomedical Imaging and Image Guided Therapy, Medical University of Vienna, Vienna, Austria

**Supplemental Tables**

**Supplemental Table 1:** Antihypertensive medication at baseline and follow-up is listed for each patient. The last columns indicates whether dosage or number of antihypertensive agents was reduced after 12 weeks of treatment

|  | **Antihypertensive Medication at Baseline** | **Antihypertensive Medication after 12 weeks metyrapone treatment** | **Reduction yes/no** |
| --- | --- | --- | --- |
| 1 | Amlodipine 10mg 0-0-1 Enalapril 20mg 1-0-0  Hydrochlorothiazide 12.5mg 1-0-0 | Amlodipine 10mg 0-0-1 Enalapril 20mg 1-0-0  Hydrochlorothiazide 12.5mg 1-0-0 | no |
| 2 | Amlodipine 5 mg 1-0-0 Candesartan cilexetil 16mg 1-0-0 Nebivolol 5mg 1-0-0 | Amlodipine 5 mg 1-0-0 Candesartan cilexetil 16mg 1-0-0 Nebivolol 5mg 1-0-0 | no |
| 3 | No antihypertensive medication | No antihypertensive medication | no |
| 4 | Amlodipine 5 mg 1-0-0 Carvedilol 25mg 1-0-1 | Amlodipine 5 mg 1-0-0 Carvedilol 25mg 0-0-1 | yes |
| 5 | Candesartan cilexetil 8mg 1-0-0 | No antihypertensive medication | yes |
| 6 | No antihypertensive medication | No antihypertensive medication | no |
| 7 | No antihypertensive medication | No antihypertensive medication | no |
| 8 | Candesartan cilexetil 16mg 1-0-0 Bisoprolol 5mg 1-0-0 Lercanidipine 10mg 1-0-1 | Candesartan cilexetil 16mg 1-0-0 Bisoprolol 5mg 1-0-0 Lercanidipine 10mg 1-0-1 | no |
| 9 | Doxazosin 4mg 1-0-0 Amlodipine 10mg 1-0-0 Valsartan 160 mg 1-0-0 Bisoprolol 10mg 0-0-1 | Doxazosin 4mg 1-0-0 Amlodipine 10mg 1-0-0 Valsartan 160 mg 1-0-0 Bisoprolol 10mg 0-0-1 | no |
| 10 | Candesartan 8mg 0-0-1/2 | No antihypertensive medication | yes |
| 11 | Amlodipine 5mg 1-0-1 Candesartan cilexetil 32mg 1-0-0 Hydrochlorothiazide 12.5mg 1-0-0 Carvedilol 25 mg 1-0-1 | Amlodipine 5mg 0-1-0 Candesartan cilexetil 32mg 1-0-0 Hydrochlorothiazide 12.5mg 1-0-0 | yes |
| 12 | Candesartan cilexetil 16 mg 1-0-1 Hydrochlorothiazide 12.5mg 1-0-0 | Candesartan cilexetil 16 mg 1-0-1 Hydrochlorothiazide 12.5mg 1-0-0 | no |
| 13 | Lercanidipine 10 mg 0-0-1 Valsartan 160 mg 1-0-0 Hydrochlorothiazide 12.5 mg 1-0-0 | Lercanidipine 10 mg 0-0-1 Valsartan 160 mg 1-0-0 Hydrochlorothiazide 12.5 mg 1-0-0 | no |
| 14 | Candesartan cilexetil 8 mg 1-0-0 | Candesartan cilexetil 8 mg 1-0-0 | no |
| 15 | Ramipril 2.5 mg ½-0-0 | Ramipril 2.5 mg ½-0-0 | no |

**Supplemental Table 2:** (A) A robust linear regression model was applied to evaluate association with percentage change of hepatic lipid content after treatment. T-values were converted using the formula: 2*(1-pt(abs(t-value)),df=8)). Urinary free cortisol, fasting glucose and percentage of body fat were included in the analysis. (B) Spearman’s correlation coefficient analysis in-between covariates. (C) Variance inflation factor analysis for determination of multicollinearity.

**A**

| **Coefficients** | **Estimate** | **Std. Error** | **p-value** |
| --- | --- | --- | --- |
| Intercept | 110·172 | 19·592 | 0·00030 |
| 24h urinary free cortisol | 0·098 | 0·051 | 0·090 |
| Fasting glucose | -0·560 | 0·123 | **0**·**0019** |
| Body fat (%) | -0·626 | 0·236 | **0**·**029** |

**B**

| **Coefficients** | **24h urinary free cortisol** | **Fasting glucose** | **Body fat mass (%)** |
| --- | --- | --- | --- |
| 24h urinary free cortisol | 1·000 | -0·141 | -0·692 |
| Fasting glucose | -0·141 | 1·000 | 0·177 |
| Body fat (%) | -0·692 | 0·177 | 1·000 |

**C**

| **Coefficients** | **24h urinary free cortisol** | **Fasting glucose** | **Body fat mass (%)** |
| --- | --- | --- | --- |
| Variance inflation factor | 1·626 | 1·055 | 1·566 |
|  |  |  |  |

**Supplemental Figures**

**Supplemental Figure 1:** Comparison of body composition before and after 12 weeks of metyrapone treatment

**Figure Legend supplemental Figure 1:** For each patient (A) waist circumference, (B) BMI, (C) total body fat mass and (D) total body fat free mass were compared after 12 weeks of metyrapone treatment using the Wilcoxon signed-rank test. No significant differences were found regarding waist circumference (p = 0·78), BMI (p = 0·36), total body fat mass (p = 0·39), and total body fat free mass (p = 0·93).

**Supplemental Figure 2:** Comparison of insulin sensitivity markers before and after treatment. Abbreviations: HOMA-IR, Homeostatic Model Assessment of Insulin Resistance; OGIS_120, Oral Glucose Insulin Sensitivity index at 120 minutes

**Figure Legend supplemental Figure 3:** (A) OGIS_120 and (B) HOMA-IR were compared at baseline and follow-up using Wilcoxon signed-rank tests. No significant differences were determined (A, p=0·069 and B, p = 0·14).

**Supplemental Figure 3:** Comparison of adrenal hormone parameters before and after metyrapone treatment, ACTH, adrenocorticotropic hormone; 11-DOC, 11-deoxycortisol;17-OH-progesterone, 17-hydroxyprogesterone

**Figure Legend supplemental figure 3:** Hormonal adaption after 12 weeks of treatment. Wilcoxen signed-rank tests were applied, and a significant increase was observed for ACTH (p = 0·010), 17-hydroxyprogesterone(p=0·020), 11-deoxycortisol (p= 0·00010) and androstenedione levels (p = 0·00010).
