# Supplementary material for: Metabolic effects of metyrapone treatment in patients with mild autonomous cortisol secretion: a prospective proof-of-concept trial
Source: eClinicalMedicine. 2026 Feb 6;92:103775. doi: 10.1016/j.eclinm.2026.103775 (PMC12907678; doi:10.1016/j.eclinm.2026.103775)
Supplement: Study Protocol [file mmc2.pdf]

Investigating cardiometabolic risk factors and changes in chronobiology patterns in patients with autonomous adrenal cortisol secretion – a pilot study

EudraCT number: 2022-000161-40
Version: 4
Date: 18.03.2023

Principal Investigator:
Priv. Doz. Dr. Peter Wolf, PhD
Division of Endocrinology and Metabolism
Department of Medicine IIII
Medical University of Vienna

Co-Investigators:
Medical University of Vienna, Division of Endocrinology and Metabolism, Department of Medicine III, Medical University of Vienna: Michael Leutner, Martin Krssak, Michael Krebs, Clemens Baumgartner, Hannes Beiglböck, Helene Schernthaner-Reiter, Greisa Vila, Alexandra Kautzky-Willer

University of Vienna, Center for Molecular Biology, Platform Rhythms of Life: Kristin Tessmar-Raible, Lakshmi Kalathinkunnath

University of Vienna, Faculty of PsychologyDepartment of Cognition, Emotion, and Methods in Psychology: Helmut Leder

Confidentiality Statement

The information contained in this document, especially unpublished data, is the property of the sponsor of this study. It is therefore provided to you in confidence as an Investigator, potential Investigator, or consultant, for review by you, your staff, and an Independent Ethics Committee or Institutional Review Board. It is understood that this information will not be disclosed to others without written authorization from the principal investigators except to the extent necessary to obtain informed consent from those persons to whom the study drug may be administered.

Ethics and Good Scientific Practice

The Investigator will ensure that this study is conducted in full conformance with the principles of the "Declaration of Helsinki" (revised in 2013) and with the laws and regulations of the country in which the clinical research is conducted. The Investigator of the clinical trial shall guarantee that only appropriately trained personnel will be involved in the study. All studies must follow the ICH GCP Guidelines. In other countries in which GCP Guidelines exist, the Investigators will strictly ensure adherence to the stated provisions. Therefore, this study follows the EU Directive embedded in the Austrian drug act.

**Test drug (IMP) and Pharmaceutical Company**	Metyrapone, HRA Pharma (Metycor ®)
**Document type**	Clinical study protocol
**Sponsor (contact)**	Medical University of Vienna (Peter Wolf)
**Study phase**	Phase IV

Protocol approval:

Priv. Doz. Dr. Peter Wolf, PhD
(Sponsor and Principal investigator)

Background: Autonomous cortisol secretion (ACS) is frequently present in patients with an adrenal incidentaloma. It is associated with an increased mortality, mainly because of cardiovascular diseases. So far, ACS is not a clear-cut indication for adrenal surgery leaving a large number of high-risk patients without established treatment. Furthermore, cortisol is a key mediator involved in the synchronization of circadian signals. We therefore hypothesize that (i) altered hypothalamus-pituitary-adrenal (HPA) axis signaling in ACS is characterized by a disease specific chronobiology pattern and that (ii) the restoration of a physiologic cortisol secretion pattern by metyrapone treatment improves established cardiometabolic risk factors.

Aims: We aim to perform a cardiometabolic characterization of patients suffering from autonomous cortisol secretion and to identify alterations in their chronobiology profile (Aim I and II). Furthermore, we aim to investigate the impact of a treatment by metyrapone in the evening on cardiometabolic risk factors in patients with autonomous adrenal cortisol secretion (Aim III).

Methods: Patients with and without ACS will be recruited for cross-sectional analysis. Cardiovascular and metabolic phenotyping will be performed by frequently sampled oral glucose tolerance tests to estimate insulin sensitivity and – secretion. ^{1}H magnetic resonance spectroscopy and imaging will be performed to assess subcutaneous, visceral and ectopic fat stores, as well as cardiac function. Furthermore, blood will be tested for systemic inflammation, hypercoagulability and the renin-angiotensin-aldosterone system activity. The chronobiology profile will be evaluated by standardized questionnaires together with frequent body temperature measurements. Furthermore, analyses of the peroxiredoxin oxidation cycles from red blood cells and circadian clock transcript changes from peripheral blood mononuclear cells will be performed. In a subgroup of 15 patients with ACS these examinations will be performed in a longitudinal, open label, single arm trial before and 12 weeks after a treatment by metyrapone 500 mg at 6 p.m. and 250 mg at 10 p.m.

Clinical relevance: Despite its high prevalence, treatment of ACS is controversial. Here we investigate short-term effects of a pharmacological restoration of the circadian cortisol rhythm by metyrapone. In addition, the identification of a disease specific chronobiology profile might help to diagnose patients with disturbed HPA signaling at an early stage before the development of metabolic complications.

1. Background:

Endogenous hypercortisolism in Cushing's syndrome and autonomous adrenal cortisol secretion (ACS) is associated with an increased morbidity and mortality, mainly because of cardiovascular diseases (1, 2). ACS is defined as the presence of inappropriate alterations in the hypothalamus-pituitary-adrenal (HPA) axis signaling including impaired circadian lowering of cortisol secretion during nighttime in patients with an adrenal incidentaloma, who do not fulfill diagnostic criteria for Cushing's syndrome and do not have the classic clinical and metabolic signs of cortisol excess.

Whereas diagnosis and therapy for Cushing's syndrome is well-established (3), optimal treatment for patients with ACS is controversially discussed (4). This is of special interest, since ACS is observed much more frequently in routine clinical care. About one third of patients with an adrenal incidentaloma, which can be incidentally diagnosed in approximately 10% of the general population, present a dysregulation of adrenal cortisol secretion (5).

An unphysiological circadian cortisol rhythm with elevated cortisol levels in the evening and during the night is one of the earliest signs of impaired HPA axis signaling (6). Late-in-the-day cortisol exposure was shown to modulate insulin sensitivity in skeletal muscle (7) and increases blood pressure and inflammation parameters (8). This might play a detrimental role in the development of cardiovascular diseases in patients with ACS.

So far, in contrast to Cushing's syndrome, ACS is not a clear-cut indication for adrenal surgery leaving a large number of high-risk patients without established treatment. Metyrapone is a selective 11b-hydroxylase inhibitor and is frequently used in the medical treatment of Cushing's syndrome because of its rapid effects (9). Evening doses of Metyrapone were recently shown to reset physiological day/night rhythms of cortisol by lowering nocturnal cortisol exposure, without affecting morning cortisol levels due to the short duration of action of the drug (10). However, whether this results in improvements of cardiovascular risk factors and glucose- and lipid metabolism is currently unknown.

2. Hypothesis:

We hypothesize that autonomous cortisol secretion is associated with an adverse cardio-metabolic risk profile, which will be improved by treatment with evening doses of metyrapone.

3. Aims:

Aim 1: Cardiometabolic phenotyping of patients suffering from autonomous cortisol secretion: An important role of various cardiovascular risk factors has been reported in patients with endogenous hypercortisolism: Glucose and lipid metabolism are directly worsened by cortisol action (11), which might be associated with increased ectopic lipid deposition in the liver and skeletal muscle, since this is a strong and highly sensitive predictor for insulin resistance (12). Besides these metabolic complications, heart function and morphology is altered in patients with Cushing's disease characterized by myocardial hypertrophy and reduced stroke volume (13) and increased pericardial fat mass (14, 15). This might be mediated directly by cardiotoxic effects of hypercortisolism, or indirectly by hypertension, systemic inflammation or increased sensitivity to catecholamines and angiotensin 2 (9). In addition, cortisol excess is associated with a state of hypercoagulability, which contributes to the development of cardiovascular events (16). In order to cover the diverse spectrum of potential underlying pathomechanisms, study participants will undergo a thoroughly investigation of metabolic, cardiovascular, inflammatory and hemostatic risk factors.

Aim 2: Identifying a disease specific chronobiology profile in patients with altered hypothalamus-pituitary-adrenal (HPA) axis signaling: Cortisol is a key mediator for the synchronization of clock genes, as well as the rhythmic expression of circadian signals in almost all tissues. We therefore hypothesize that altered HPA axis signaling in ACS is characterized by a specific chronobiology pattern. We further hypothesize that these alterations in circadian signaling occur at an early stage of impaired adrenal cortisol secretion, since changes in HPA axis activity can be observed immediately after shift work and as a result of jetlag (17). Identification of a characteristic chronobiology pattern might enable to identify even subtle alterations in HPA axis and might therefore offer a precise and highly sensitive diagnostic tool for

the detection of ACS. Potential associations of altered chronobiological pattern with cardiometabolic risk factors might improve the individual risk assessment and might facilitate personalized therapeutic strategies.

Aim 3: Investigating the impact of a treatment by metyrapone on cardiometabolic risk factors in patients with autonomous adrenal cortisol secretion: Based on the crucial role of abnormal circadian cortisol rhythm in the development of cardiovascular complications, we aim to investigate the effects of a pharmacological restauration by metyrapone. Metyrapone is a short acting, highly selective inhibitor of the 11b-hydroxylase and frequently used for the medical treatment of Cushing’s syndrome (9). Administration of Metyrapone 500 mg at 6 p.m. and 250 mg at 10 p.m. was previously shown to normalize the circadian cortisol rhythm (10). We propose that lowering evening cortisol levels improves metabolic, cardiovascular, inflammatory and hemostatic risk factors identified in aim 1, with only minimal risk for adrenal insufficiency due to the short half-life of the drug. We therefore aim to investigate patients before and 12 weeks after initiation of medical treatment by metyrapone in an open-label single arm study design.

4. Outcome parameters:

Primary outcome parameter:

- Hepatic lipid content (HCL) assessed by ^{1}H magnetic resonance spectroscopy

Secondary outcome parameters:

- insulin secretion and sensitivity based on validated indices (HOMA_IR, CLIX)
- subcutaneous and visceral fat mass, body composition
- Systolic and diastolic heart function, epicardial and pericardial fat mass
- ectopic lipid deposition in the skeletal muscle and the myocardium
- standardized blood pressure measurements (blood pressure for 24h hours and Schellong tests)
- parameters of systemic inflammation (CRP, Il-6, PAI-1, TNF-alpha, homocysteine, leucocytes, monocytes)
- parameters of the Renin-Angiotensin-Aldosterone system (RAAS) activation (RAAS FINGERPRINT ®) (18)

- hypercoagulability assessment by thrombin generation assays (TECHNOTHROMBIN ®)
- standardized clinical chronotype evaluation by Munich ChronoType Questionnaire and psychological testing
- body temperature measurements during the day by an actimetry watch
- analyses of peroxiredoxin oxidation cycles from red blood cells (19),
- circadian clock transcript changes from peripheral blood mononuclear cells (20),
- combined untargeted/targeted LC-MS/MS based approaches
- concentrations of testosterone and adrenal androgen precursors
- gender specific differences

5. **Methods**:

Study design:

Working plan A (WPA):

Cardiometabolic phenotyping:

To address aim 1 we will perform a cross-sectional study in patients, who are in routine care at the endocrine outpatients' clinic for the hormonal work up of an adrenal adenoma. 25 patients with an ACS will be recruited and compared to 25 patients of comparable age and sex with a sufficient suppression of cortisol < 1.8 ug/dl following a 1 mg dexamethasone suppression test.

Experiments will be performed in the morning after an overnight fast. Subjects have to avoid intense physical exercise and consumption of alcoholic beverages for at least 48 hours previous to study related experiments.

^{1}H magnetic resonance (MR) spectroscopy measurements will be performed in high field 3T whole body MR system (Magnetom, Siemens Healthcare, Erlangen, Germany) equipped with appropriate surface coils. Ectopic fat in the liver, the skeletal muscle and the myocardium will be assessed by the single voxel localized ^{1}H MRS method (41).

After the MR measurements a frequently sampled oral glucose tolerance test will be performed and surrogate parameters of insulin secretion and sensitivity will be

calculated based on validated indices (HOMA_IR, CLIX, OGIS, IGI, Disposition Index, Adaptation Index) (21).
Additionally, blood will be drawn at baseline for the measurement of parameters of systemic inflammation (CRP, Il-6, PAI-1, TNF-alpha, homocysteine, leucocytes, monocytes), the renin-angiotensin-aldosterone system (RAAS FINGERPRINT ®) and hypercoagulability (TECHNOTHROMBIN ®).
To estimate diurnal cycles of cortisol secretion salivary cortisol will be drawn at 8:00 a.m. and midnight.

To address aim 2, additional blood will be drawn to analyze the peroxiredoxin oxidation cycles from red blood cells (19), as well as the circadian clock transcript changes from peripheral blood mononuclear cells (21).
In addition, blood will be stored for combined untargeted/targeted LC-MS/MS based metabolomic/proteomic approaches.

Overall, 125 ml blood will be drawn in total for all biochemical analyses of WPA.

A systematical assessment of the chronotype will be performed by using the Munich ChronoType Questionnaire. Furthermore, the following psychological tests will be applied:

- Measurement of vigilant attention and impulsivity (by means of the Psychomotor Vigilance Task)
- Implicit association of concepts (by the Implicit Association Test)
- Theory of mind (by Reading the mind in the eyes test)
- Ability to match unfamiliar faces (by the Glasgow Face Matching Test)

The performance of all psychological tests for the comprehensive evaluation of the chronotype will take about 2 hours. In order to avoid prolonged fasting periods, study participants will be allowed to have breakfast before completing the questionnaires.

<u>Assessment of the chronotype:</u>
This part of the study will be performed online. <u>Details regarding the online platform are as follows:</u>
The experiments will be done via a web-based platform named ‘Labvanced’.

Labvanced consists of various functions to create, run, and share online experiments and psychological studies directly in the browser (https://www.labvanced.com/). For data collection, the current study will use password protection such that the participants with the correct password (which will be given to them along with the instructions for the experiment) can only open and start the experiment. The data collected from the experiments will not be processed by any external providers and will be handled only by the authorized people concerned with the project.

Regarding data security and privacy, the documentation provided by Labvanced states that their servers ensure the highest security standards in a professional server center managed by the Hetzner GmbH (for more details, kindly refer to this URL: https://www.labvanced.com/static/docs/TOM_Scicovery_German.pdf).

The data stored in the labvanced server is under full control of the users; and even though the server keeps a 7-day backup in case of unintentional data deletion, after that period, all the data will be deleted. Summary of the key points regarding the privacy statement can be found in the following url:

https://www.labvanced.com/privacy.html .

Labvanced has been used by several psychology-based studies, and the following link provides a list of publications citing labvanced:

https://docs.google.com/spreadsheets/d/1xrYwfxtPCs1OpafTGLc30fbRz8BxNK9zZ-woyvNK0qQ/edit#gid=0 .

The following experiments will be performed:

1. Munich Chronotype Questionnaire (MCTQ) (26) (max. 10 minutes): This survey includes topics interlinking the sleep-wake schedule and work schedule of the participants, as well as questions on the anxiety and stress levels of the participants. The MCTQ asks participants questions related to their sleep and wake up times, answers of which will be assigned to specific variables which will then be used to calculate the chronotype, which is an established way of analyzing the rhythmicity of individuals (22).

The following parameters are included in the MCTQ:

- Sleep and wake up times of the participant
- Work schedules of the participant

- Time required and type of vehicle used (enclosed or non-enclosed) for commute to and from work for the participant
- Time spent outdoors by the participant
- Approximate amounts of stimulants (coffee, tea, cigarettes, etc.) consumed by the participant
- Time slots wherein the participant has major meals in a day
- Type of exercise/workout routines that the participant has, its duration and regularity
- Sleep quality of the participant
- Diseases and other medical conditions of the participant (apart from AI)
- Time zone difference if the participant has travelled to another country in the past two months
- Family status of the participant (whether the participants have/are expecting children, but also, whether they have pets, etc.)
- Current stress and anxiety levels of the participant as measured through five-point scales
- Positive and Negative Affect measured with 10 item scales (words such as ‘Inspired’, ‘Upset’, ‘Nervous’, etc.), with each item rated on a five-point scale.

Based on this MCTQ the individual chronotype can be calculated as mid-sleep time on free days corrected for sleep debt on work days (MSFsc) and can be categorized in 3 main types and 7 subtypes. To exemplify, if an individual starts sleeping at midnight, wakes up at 8am and requires no correction for sleep debt, the MSFsc– i.e. chronotype– is 4. It should also be said that this is a very established method to access the circadian chronobiology in humans (e.g. Epidemiology of the human circadian clock by Institute for Medical Psychology, University of Munich doi:10.1016/j.smrv.2007.07.005).

The subdivision of chronotype will follow the standardized recommendations of Prof.Till Roennerberg, Institut für Medizinische Psychologie, Zentrum für Chronobiologie on this matter, who established this test (for details also see https://de.wikipedia.org/wiki/Chronotyp#cite_note-LMU2010-1).

After completion of the questionnaires, the participants will be directed to a set of cognitive and psychology-based tasks.

2. The following cognitive and psychology-based tasks will be performed during the study:

A) The Psychomotor Vigilance Task (max. 10 minutes):

The Psychomotor vigilance task (PVT) measures the attention and alertness of an individual. It is widely used to test sustained attention in the circadian domain (23). The PVT test measures sustained or vigilant attention by recording response times (RT) to visual or auditory stimuli that occur at random inter-stimulus intervals. In the current study, we plan to use visual stimuli.

B) The Glasgow Face Matching Test (max. 10 minutes):

The Glasgow Face Matching Test (GFMT) is used to test the participant's ability to match unfamiliar faces. This test usually asks participants to match two images in the same view, but taken with different cameras. The GFMT that we are planning to use in this study, called the GFMT2, is a revised version of the GFMT, which is slightly more difficult than the earlier version, since it includes variation in head angle, pose, expression, and subject to camera distance (24).

C) Reading the Mind in the Eyes Test (max. 10 minutes):

Reading the mind in the eyes test (RMET) assesses the ability of a person to recognize complex mental states as expressed by the human eyes, and is an adult test of social sensitivity. Participants are given photographs of different pairs of eyes and are asked to judge what mental state that the people in the photograph is in, by choosing one of four options of words that they think best describes what the person in the photograph is experiencing or thinking. There are 'correct' responses to the test which are based on majority responses from a number of expert judges from a healthy population (25).

D) The Implicit Association Test (max. 10 minutes):

The Implicit Association Test is one of the most frequently used measurements of implicit associations – it measures strengths of differential association of concepts with

attributes. In this study, we plan to measure the association of brightness with positivity, following the works of Specker at al (26).

E) The Visual Aesthetic Sensitivity Test (max. 10 minutes):

The Visual Aesthetic Sensitivity Test (VAST) is a test wherein participants are shown one pair of seemingly likely paintings at a time, and are asked which of the two pairs seem more aesthetically pleasing than the other. Each pair of artworks are very similar, black and white and abstract, and are designed by an artist, taking to control in regards to content, color and complexity and largely removing any higher associations or meanings. One in each pair was changed to be less 'aesthetically pleasing' than the other by incorporating certain intentional design faults. The stimuli pairs were rated by art experts and those pairs chosen that showed a certain amount of shared agreement with the goal of having one image of each pair appear objectively superior in terms of its aesthetic quality or beauty, featuring its symmetry, balance, etc. (27).

An up-to-date version of the experiment is provided in the link below for your reference; kindly note that the password for the same is **chrono123**

https://www.labvanced.com/player.html?id=31460

The participants have to click on a similar link, enter a prescribed password and go through the series of questionnaires and experiments.

Actimetry watch:

Besides performing the MCTQ and the cognitive and psychology-based tasks, included participants will be asked to wear an actimetry watch during a period of at most three months. Again, this is a very established procedure for the study of human chronobiology (e.g. see Science Advances 7(5):eabe0465 DOI:10.1126/sciadv.abe0465)

All measurements registered by the watch will be analyzed at the end of each period by connecting the watch with the suitable software. There is no wireless data transfer during the study period. All data is stored only on the laptop, who is secured with a password. Of note, only the study specific subject ID, but no other identifying variables

will be entered in the actimetry software to ensure the pseudonymization of all patients' data.

Parameters derived from the actimetry watch:

Sl No.	Parameter	Time of measurement
1.	Sleep Onset	Continuous measurement for a period of no more than 3 months.
2.	Sleep Duration	
3.	Sleep Efficiency	
4.	Activity	
5.	Temperature	
6.	Ambient light exposure	

Working plan B (WPB):

A subgroup 15 consecutive patients, who fulfill the diagnostic criteria of an ACS, will undergo a 12 weeks treatment period with metyrapone. Metyrapone will be administered with 500 mg at 6 p.m. and 250 mg at 10 p.m., which was previously shown to normalize the circadian cortisol rhythm (10) with only limited risk of an adrenal insufficiency because of the short half-life of the drug.

In these patients, all measurements of WPA will be performed at baseline and after 12 weeks of treatment with metyrapone.

A total of 125 ml blood will be drawn at study inclusion and after 12 weeks of treatment. Therefore, 250 ml blood will be drawn overall during the 3 months study period.

Additionally, to the above-mentioned measurements, the usage of an actimetry watch during the study period will help to monitor the sleep-wake cycles, temperature fluctuations and activity of the individuals to evaluate changes over time.

A clinical evaluation at the endocrine outpatients' clinic will be performed after 2, 4 and 8 weeks of treatment to assess the compliance of study drug intake and clinical tolerance, as well as the need for adaptations in concomitant treatment. Besides blood

pressure, heart rate and body weight, clinical signs of adrenal insufficiency (fatigue, orthostatic hypotension, hypoglycemia) will be recorded systematically. Blood will be drawn only in case of clinical need.

Patients' population:

ACS group: 25 patients with an ACS diagnosed during the routine hormonal workup of an adrenal incidentaloma, who are in regular care at the Division of Endocrinology and Metabolism, Medical University of Vienna, will be included. Contact exists to > 500 patients with an adrenal incidentaloma based on recent registry analysis. Patients in routine care at the endocrine outpatients' clinic will be invited to participate in study related activities. In addition, eligible patients from the registry will be contacted by a letter, starting with the most recent ones.

Inclusion criteria for the ACS group:

- Age > 18 years
- History of an adrenal adenoma
- morning cortisol > 1.8 ug/dl following 1 mg dexamethasone suppression test
- No classical clinical features associated with the presence of Cushing's syndrome

Control group: 25 patients with an adrenal incidentaloma, in which endogenous hypercortisolism was excluded during the routine hormonal workup at the Division of Endocrinology and Metabolism, Medical University of Vienna, will be included as control group.

Inclusion criteria for the control group:

- Age > 18 years
- History of an adrenal adenoma
- morning cortisol of ≤ 1.8 ug/dl following 1 mg dexamethasone suppression testing

General exclusion criteria:

- HbA1c > 8 % and/or treatment with insulin therapy

- uncontrolled hypertension (RR > 170/110 mmHg; treatment with no more than 4 antihypertensive drugs)
- previous treatment with glucocorticoids within the last 3 months
- concomitant treatment with drugs affecting HPA signaling or CYP3A4 metabolism
- adrenal tumor with radiological criteria suspicious for malignancy
- chronic kidney disease (eGFR < 45 ml/min)
- liver disease (ASAT / ALAT > 3 x ULN)
- pregnancy or breast feeding
- general MR contraindications (claustrophobia, metal devices or other magnetic material in the body which will be hazardous for MR investigation

Of note, all biochemical work-up of hormonal activity of the adrenal incidentaloma will be performed at the endocrine outpatients' clinic independently of study related activities. Only patients, who already completed their hormonal analyses will be asked to participate in the study.

6. Withdrawal and replacement of subjects

Criteria for withdrawal

Subjects may prematurely discontinue from the study at any time. Premature discontinuation from the study is to be understood when the subject did not undergo End of Study (EOS) examination and / or all pivotal assessments during the study.
Subjects must be withdrawn under the following circumstances:

- at their own request
- if the investigator feels it would not be in the best interest of the subject to continue
- if the subject violates conditions laid out in the consent form / information sheet or disregards instructions by the study personal

In all cases, the reason why subjects are withdrawn must be recorded in detail in the CRF and in the subject's medical records. Should the study be discontinued prematurely, all study materials (complete, partially completed and empty CRFs) will be retained.

Replacement policy: Dropouts are not included in the sample size and will be replaced by allocation of the next free subject number.

Follow-up of patients withdrawn from the study

In case of premature discontinuation after study drug intake, the investigations scheduled for the EOS visit will be performed within 7 days after study drug discontinuation. The subjects will be advised that participation in these investigations is voluntary. Furthermore, they may request that from the time point of withdrawal no more data will be recorded and that all biological samples collected in the course of the study will be destroyed.

Premature termination of the study

The investigators have the right to close this study at any time. The ethics committee and the competent regulatory authority will be informed within 15 days of early termination.

The trial or single dose steps will be terminated prematurely in the following cases:

- If adverse events occur which are so serious that the risk-benefit ratio is not acceptable.
- If the number of dropouts is so high that proper completion of the trial cannot realistically be expected.

7. Study medication

Active agent and characteristics: Metyrapone

Trade name of the agent: Metycor 250 mg

Supply Chain: Metycor will be provided by HRA pharma. The drug will be stored in accordance with the prescribing information.

IMP administration and handling: Metycor will be taken orally with a dose of 500 mg at 6 p.m. and 250 mg at 10 p.m.

Drug Accountability and assessment of subjects' compliance: Drug accountability and subjects' compliance will be directly observed by the investigators. Additionally, batch numbers will be recorded in the CRF.

Concomitant medication: Concomitant medication will be registered at every study visit.

Randomization and blinding: This study is designed as an open label, single armed trial. No randomization and blinding will be performed.

8. Potential Risks

Metyrapone is a selective inhibitor of the 11-b hydroxylase and is frequently used in the medical treatment of Cushing's syndrome because of its rapid effects (9).
Being generally well tolerated minor, but common side effects include **gastrointestinal side effects** including diarrhea, nausea and vomiting, as well as **fatigue, dizziness and headache**, which are all rapidly reversible after interruption of the drug.
Furthermore, side effects of **hyperandrogenemia** can occur in women, resulting rarely in hirsutism and alopecia. Hyperandrogenemia spontaneously resolves after interruption of metyrapone.
It is important to note that these side effects appear to be largely depending on the daily dose of metyrapone, as well as on the severity of hypercortisolism before treatment of Cushing's syndrome. We therefore assume, that the risk of these side effects is limited in our study, using small daily doses of metyrapone (750 mg per day) in patients with only mild hypercortisolism.
The most dangerous side effect of metyrapone is **adrenal insufficiency**, which can lead to life threatening adrenal crisis in situations of acute stress. Metyrapone is a short-acting steroidogenesis inhibitor and doses given at 6 p.m. and 10 p.m. were shown to lower only cortisol levels in the evening and during the night, without affecting cortisol levels in the morning and afternoon (10). We therefore assume that the risk of adrenal insufficiency is very limited with our treatment regime. However, all study participants will be educated on earliest clinical signs and symptoms of adrenal insufficiency. They will be systematically instructed in the supplementation of Hydrocortisone by tablets in case of the suspicion of an adrenal insufficiency and by subcutaneous injection of hydrocortisone in case of the suspicion of an adrenal crisis. In addition, clinical signs and symptoms of adrenal insufficiency will be assessed systematically at every study visit.

9. Safety Definitions and Reporting Requirements

Summary of known and potential risks of the study drug

Please find this information provided in the expert information, which will be attached.

Definition of adverse events

An AE is any untoward adverse change from the subject's baseline condition, i.e., any unfavorable and unintended sign including an abnormal laboratory finding, symptom or disease, which is considered to be clinically relevant by the physician that occurs during the course of the study, whether or not considered related to the study drug.

Adverse events include:

- Exacerbation of a pre-existing disease.
- Increase in frequency or intensity of a pre-existing episodic disease or medical condition.
- Disease or medical condition detected or diagnosed after study drug administration even though it may have been present prior to the start of the study.
- Continuous persistent disease or symptoms present at baseline that worsen following the start of the study.
- Lack of efficacy in the acute treatment of a life-threatening disease.
- Events considered by the Investigator to be related to study-mandated procedures.
- Abnormal assessments, e.g. physical examination findings, must be reported as AEs if they represent a clinically significant finding that was not present at baseline or worsened during the course of the study.
- Laboratory test abnormalities must be reported as AEs if they represent a clinically significant finding, symptomatic or not, which was not present at baseline or worsened during the course of the study or led to dose reduction, interruption or permanent discontinuation of study drug.

Adverse events do not include:

- Pre-planned interventions or occurrence of endpoints specified in the study protocol are not considered AE´s, if not defined otherwise (eg.as a result of overdose)
- Medical or surgical procedure, e.g., surgery, endoscopy, tooth extraction, transfusion. However, the event leading to the procedure is an AE. If this event is serious, the procedure must be described in the SAE narrative.
- Pre-existing disease or medical condition that does not worsen.

- Situations in which an adverse change did not occur, e.g., hospitalizations for cosmetic elective surgery or for social and/or convenience reasons.
- Overdose of either study drug or concomitant medication without any signs or symptoms. However, overdose must be mentioned in the Study Drug Log.

Serious Adverse Events (SAEs)

A Serious Adverse Event (SAE) is defined by the International Conference on Harmonization (ICH) guidelines and GCP guidelines as any AE fulfilling at least one of the following criteria:

- Results in deaths.
- Life-threatening – defined as an event in which the subject was, in the judgment of the Investigator, at risk of death at the time of the event;
- Requiring subject's hospitalization or prolongation of existing hospitalization
- Resulting in persistent or significant disability or incapacity (i.e., a substantial disruption of a person's ability to conduct normal life functions).
- Congenital anomaly or birth defect.
- Is medically significant or requires intervention to prevent at least one of the outcomes listed above

Life-threatening refers to an event in which the subject was at risk of death at the time of the event. It does not refer to an event that hypothetically might have caused death if it were more severe.

Important medical events that may not immediately result in death, be life-threatening, or require hospitalization may be considered as SAEs (optional!) when, based upon appropriate medical judgment, they may jeopardize the subject and may require medical or surgical intervention to prevent one of the outcomes listed in the definitions above. This means an individual case decision.

Hospitalization – Prolongation of existing hospitalization

Hospitalization is defined as an overnight stay in a hospital unit and/or emergency room. An additional overnight stay defines a prolongation of existing hospitalization.

The following is not considered an SAE and should be reported as an AE only:

- Treatment on an emergency or outsubject basis for an event not fulfilling the definition of seriousness given above and not resulting in hospitalization.

The following reasons for hospitalizations are not considered AEs, and therefore not SAEs:

- Hospitalizations for cosmetic elective surgery, social and/or convenience reasons.
- Elective treatment of a pre-existing disease or medical condition that did not worsen, e.g., hospitalization for chemotherapy for cancer, elective hip replacement for arthritis.

SAEs related to investigational drug

Such SAEs are defined as SAEs that appear to have a reasonable possibility of causal relationship (i.e., a relationship cannot be ruled out).

Suspected unexpected serious adverse reactions (SUSARs)

SUSARs are all serious adverse reactions with **suspected** (not only possible) causal relationship to the study drug that is unexpected (not previously described in the Summary of Product Characteristics or Investigator's brochure) and serious.

Severity of adverse events

The severity of clinical AEs is graded on a three-point scale: mild, moderate, severe, and reported on specific AE pages of the CRF.

If the severity of an AE worsens during study drug administration, only the worst intensity should be reported on the AE page. If the AE lessens in intensity, no change in the severity is required.

If an AE occurs during the treatment phase, a new AE page must be filled in with the intensity observed during study drug administration.

Mild

Event may be noticeable to subject; does not influence daily activities; the AE resolves spontaneously or may require minimal therapeutic intervention;

Moderate

Event may make subject uncomfortable; performance of daily activities may be influenced; intervention may be needed; the AE produces no sequelae.

Severe

Event may cause noticeable discomfort; usually interferes with daily activities; subject may not be able to continue in the study; the AE produces sequelae, which require prolonged therapeutic intervention.

A mild, moderate or severe AE may or may not be serious. These terms are used to describe the intensity of a specific event (as in mild, moderate, or severe myocardial infarction). However, a severe event may be of relatively minor medical significance (such as severe headache) and is not necessarily serious. For example, nausea lasting several hours may be rated as severe, but may not be clinically serious. Fever of 39°C that is not considered severe may become serious if it prolongs hospital discharge by a day. Seriousness rather than severity serves as a guide for defining regulatory reporting obligations.

Relationship to study drug

For all AEs, the Investigator will assess the causal relationship between the study drug and the AE using his/her clinical expertise and judgment according to the following algorithm that best fits the circumstances of the AE:

Unrelated

- May or may not follow a temporal sequence from administration of the study product
- Is biologically implausible and does not follow known response pattern to the suspect study drug (if response pattern is previously known).
- Can be explained by the known characteristics of the subject's clinical state or other modes of therapy administered to the subject.

Unlikely

- There is a reasonable temporal relation between the AE and the intake of the study medication, but there is a plausible other explanation for the occurrence of the AE.

Possibly

- The AE has a reasonable temporal relationship with drug administration.
- The AE may equally be explained by the study subject's Clinically state, environmental or toxic factors, or concomitant therapy administered to the study subject.
- The relationship between study drug and AE may also be pharmacologically or clinically plausible.

Probably

- There is a reasonable temporal relation between the AE and the intake of the study medication, and plausible reasons point to a causal relation with the study medication.

Related

- Reasonable temporal relation between the AE and the intake of the study medication and
- there is no other explanation for the AE and
- subsidence or disappearance of the AE on withdrawal of the study medication and
- recurrence of the symptoms on restart at previous dose (only applies for re-institution of mediation).

Not assessable

- The causal relationship between the study drug and the AE cannot be judged.

Reporting procedures

A special section is designated to adverse events in the case report form. The following details must thereby be entered:

- Type of adverse event
- Start (date and time)
- End (date and time)
- Severity (mild, moderate, severe)
- Serious (no / yes)
- Unexpected (no / yes)
- Outcome (resolved, resolving, not resolved, resolved with sequelae, unknown, fatal)
- Relation to study drug (Related/ Probably/ Possibly/ Unlikely/ Not related/ Not assessable)

Adverse events are to be documented in the case report form in accordance with the above-mentioned criteria.

Reporting procedures for SAEs

In case of a serious adverse event, the Investigator has to use all supportive measures for best patient treatment. A written report is also to be prepared and should at least contain the following:

- Patient number
- Patient: sex
- The suspected investigational medical product (IMP)
- The adverse event assessed as serious
- Short description of the event and outcome

If applicable, the initial report should be followed by the Follow up report, indicating the outcome of the SAE. The reports will be submitted to the local ethics-committee, the local authorities (BASG) and to Astra Zeneca (the supplier of the study drug).

Reporting procedures for SUSARs

It must be remembered that the regulatory authorities, and in case of SUSARs which could possibly concern the safety of the study participants, also the Institutional Review Board / Independent Ethics Committee (IRB / IEC) are to be informed. Such reports shall be made by the study management and the following details should be at least available:

- Patient number
- Patient: age in years, sex
- Name of Investigator and investigating site
- Period of administration
- The suspected investigational medical product (IMP)
- The adverse event assessed as serious and unexpected, and for which there is a suspected causal relationship to the IMP
- Concomitant disease and medication
- Short description of the event:
    - Description
    - Onset and if applicable, end
    - Therapeutic intervention
    - Causal relationship
    - Seriousness criteria or reportable reason

Electronic reporting should be the expected method for reporting of SUSARs to the competent authority. In that case, the format and content as defined by the ICH E2A

Guideline should be adhered to. The latest version of MedDRA should be applied. Lower level terms (LLT) should be used.

Annual Safety Report

The Annual Safety Report will be provided by the Investigator at least once a year. This report will also be presented annually to the Independent Ethics (IEC) and to the competent authorities by the sponsor.

10. Documentation and data management

Documentation of study results

A subject screening and enrolment Log will be completed for all eligible or nor eligible subjects with the reason for exclusion.

Case report form (CRF)

For each subject enrolled, regardless of study drug initiation, a CRF must be completed and signed by Investigator or a designated sub-Investigator. This also applies to those subjects who fail to complete the study. If a subject withdraws from the study, the reason must be note on the CRF.

Case report forms are to be completed on an ongoing basis.

CRF entries and corrections will only be performed by study site staff, authorized by the Investigator. All forms should be completed and must be legible. Errors should be crossed out but not obliterated, the correction inserted, and the change initialled, and dated by the Investigator, co-Investigator or study nurse.

The entries will be checked by trained personnel (Monitor) and any errors or inconsistencies will be checked immediately.

The Monitor will collect original completed and signs CRFs at the end of the study. A copy of the completed and signed CRFs will remain on site.

Data Collection

Data collected at all visits are entered into an interactive form. The CRFs will be source documents verified following guidelines established before study onset as detailed in the Monitoring Plan. Maintenance of the study database will be performed by the designated sub-investigators. Each participant will be assigned to a study specific patient number to ensure anonymity.

Missing, Unused, and Spurious Data

All available data will be included in the data listings. Complete Case Analysis is performed on only those subjects with a complete set of primary outcome data. Subjects with any missing primary outcome data are excluded from the statistical analysis. Dropouts will be replaced. All data recorded on the CRF will be included in the data listings that will accompany the clinical study report.

Safekeeping

The Investigator will maintain adequate and accurate record to enable the conduct of the study to be fully documented and the study data to be subsequently verified (according to ICH-GCP “essential documents”). These documents will be classified in two different categories: Investigator`s file, and subject clinical source documents.

The Investigator`s file will contain the protocol/amendments, EudraCT forms, CRFs (eCRF printout), standard operation procedures (SOPs), data clarification and query forms, EC/IRB and Health Authority approval with correspondence, informed consent, drug records, staff curriculum vitae and authorization forms, screening and enrolment logs, and other appropriate documents/correspondence as per ICH/Good Clinical Practice (GCP) and local regulations. Subject clinical source documents include, but are not limited to subject hospital/ clinic record, physician`s and nurse`s notes, appointment book, original laboratory reports, ECG, X-ray, pathology and special assessment reports, consultant letters, etc.

These two categories of documents must be kept on file by the Investigator for as long as needed to comply with national and international regulations (in Austria 15 years after discontinuing clinical development or after the last marketing approval). If source documents are not durable as long as needed (e.g. ECG printouts) they must be preserved as a copy. No study document should be destroyed without prior written approval from the Department of Internal Medicine III, Division of Endocrinology and Metabolism.

When source documents are required for the continued care of the subjects, appropriate copies should be made for storing outside of the site.

Periodic Monitoring

Periodic monitoring will be performed by trained personal from the Medical University of Vienna, who will not be involved in any other study related activities. The designated monitor (Dr. Paul Fellinger) will contact and visit the Investigator regularly and will be

allowed to have access to all source documents needed to verify the entries in the CRFs and other protocol-related documents provided that subjects confidentiality is maintained in agreement with local regulations. It will be the monitor`s responsibility to inspect the CRFs at regular intervals according to the monitoring plan throughout the study to verify the adherence to the protocol ant the completeness, consistency and accuracy of the data being entered on them. The monitoring standards require full verification for the presence of informed consent, adherence to the inclusion/exclusion criteria, documentation of SAEs and the recording of the main efficacy, safety and tolerability endpoints.
The monitor will be working according to SOPs and will provide a monitoring report after each Visit for the sponsor. Depending on the quality of the data, additionally monitoring visits may be necessary according to the sponsor`s discretion. The Investigator will resolve discrepancies of the data.

Audit and Inspections
Upon request, the Investigator will make all study-related source data and records available to competent authority inspectors. The main purposes of an audit or inspection are to confirm that the rights and the welfare of the subjects have been adequately protected, and that all data relevant for safety and efficacy of the investigational product have appropriately been reported to the sponsor.

11. Ethical and Legal Aspects

Informed consent of subjects
Following comprehensive instruction regarding the nature, significance, impact and risks of this clinical trial, the patient must give written consent to participation in the study.
During the instruction the trial participants are to be made aware of the fact that they can withdraw their consent – without giving reasons – at any time without their further medical care being influenced in any way.
In addition to the comprehensive instructions given to the trial participants by the Investigator, the trial participants also receive a written patient information sheet in comprehensible language, explaining the nature and purpose of the study and its progress.

The patients must agree to the possibility of study-related data being passed on to relevant authorities.

The patients must be informed in detail of their obligations in relation to the trial participants insurance in order not to jeopardize insurance cover.

Acknowledgement/ approval of study

The Investigator will submit this protocol and any related document provided to the subject (such as subject information used to obtain informed consent) to an Ethics Committee (EC) or Institutional Review Board (IRB). Approval from the committee must be obtained before starting the study.

The clinical trial shall be performed in full compliance with the legal regulations according to the Drug Law (AMG - Arzneimittelgesetz) of the Republic of Austria.

An application must also be submitted to the Austrian Competent Authorities (Bundesamt für Sicherheit im Gesundheitswesen (BASG) represented by the Agency for Health and Food Safety (AGES PharmMed) and registered to the European Clinical Trial Database (EudraCT) using the required forms. The timelines for (silent) approval set by national law must be followed before starting the study.

Changes in the Conduct of the Study

Protocol amendments

Proposed amendments must be submitted to the appropriate CA and ECs. Substantial amendments may be implemented only after CA/EC approval has been obtained. Amendments that are intended to eliminate an apparent immediate hazard to subjects may be implemented prior to receiving CA/EC approval. However, in this case, approval must be obtained as soon as possible after implementation.

Study Termination

If the sponsor or the Investigator decides to terminate the study before it is completed, they will notify each other in writing stating the reasons of early termination. In terminating the study, the sponsor and the Investigator will ensure the adequate consideration is given to the protection of the subject interests. The Investigator, sponsor or (designated CRO on behalf of the sponsor) will notify the relevant CA and EC. Documentation will be filed in the Trial Master and Investigator Files.

Clinical Study Report (CSR)

Within one year after the final completion of the study, a full CSR will be prepared by the sponsor and submitted to the EC and the competent authority.
The Investigator will be asked to review and sign the final study report.

Insurance

During their participation in the clinical trial the patients will be insured as defined by legal requirements. The Investigator of the clinical trial will receive a copy of the insurance conditions of the 'patients insurance'. The sponsor is providing insurance in order to indemnify (legal and financial coverage) the Investigator/center against claims arising from the study, except for claims that arise from malpractice and/or negligence. The compensation of the subject in the event of study-related injuries will comply with the applicable regulations. Details on the existing patients insurance are given in the patient information sheet.

12. Prospective benefits/relevance

We suppose, that patients with ACS exhibit subclinical signs of 1) disturbed glucose and lipid metabolism, like impaired insulin tolerance, increased visceral fat mass and ectopic hepatic lipid deposition, as well as 2) cardiovascular risk factors, including higher blood pressure, diastolic dysfunction and increased left ventricular septal thickness, as well as 3) changes in chronobiology patterns. Medical restoration of a physiological circadian cortisol rhythm will normalize alterations in the individual chronobiology profile, accompanied by improvements in surrogate parameters of cardiometabolic risk.
Furthermore, the identification of a disease specific chronobiology pattern, which enables to identify even subtle alterations in HPA axis, might offer a novel diagnostic approach to select patients with ACS from non-functioning adrenal incidentaloma. This might help to diagnose patients at risk for adverse effects of unphysiological adrenal cortisol secretion at an early stage, before cardiometabolic and musculoskeletal sequelae develop. The results of this feasibility study might therefore pave the way for future, large interventional studies addressing this promising topic.

13. Feasibility

All study related activities will be performed at the Medical University of Vienna. The Division of Endocrinology has a fully equipped biomedical lab and a human research unit (Outpatient Clinic for Metabolic Research i.e. Outpatient Clinic for Metabolic Research “Chiari”). The access to the MR facility for the non-invasive measurements is warranted in the cooperation with Prof. Dr. Trattnig (MR Centre of Excellence) at the Dep. of Radiology, Medical University of Vienna. There is currently a 3 Tesla Magnetom, scanner available at the MR Centre of Excellence. The system is equipped with suitable software and coils. Performance testing of this setup has been performed extensively in several prior studies (16,21,22). Oral glucose tolerance tests will be performed at the Outpatient Clinic for Metabolic Research “Chiari” of the Division of Endocrinology and Metabolism. MR-studies will take place at the centre of excellence of high-field MR of the Medical University of Vienna. Chronobiology surrogates will be analyzed in cooperation with Prof. Kristin Tessmar-Raible, Platform Rhythms of Life, Center for Molecular Biology and Prof. Helmut Leder, Faculty of Psychology, University of Vienna.

14. Additional information:

Place of work and cooperation: This study will be performed as a single center study at the Medical University of Vienna in cooperation of the Division of Endocrinology and Metabolism, Department of Medicine III and the MR Centre of Excellence.

Time schedule: All study related activities are planned to be completed within 3 years, starting from 06/2022.

Sample size calculation:

Up to now there are no studies on hepatic lipid content assessed by 1H magnetic resonance spectroscopy in patients suffering from autonomous cortisol secretion. Therefore this study has to be considered as a pilot study.

However, evidence from literature and clinical experience suggests that the prevalence of insulin resistance and visceral obesity is increased in this patient population (2). As these factors are both well known to be associated with higher visceral and ectopic fat mass, we estimate that HCL might be about 25% higher in patients with ACS compared to controls. Based on an average HCL of 5 ± 2.5 % fat in a comparable elderly, overweight/obese, sedentary population, which we could observe in our previously

published control population (28) a sample size of 25 patients per group is necessary to detect between group differences to achieve $\alpha < 0.05$ and $\beta > 0.80$ in cross-sectional analysis (WPA).
For the interventional part (WPB), we expect a normalization of HCL comparable to the control cohort and therefore a reduction of about 25%. Therefore, a sample size of 15 patients is required to achieve $\alpha < 0.05$ and $\beta > 0.80$ in longitudinal analysis.
We expect 2 dropouts in WPA and WPB. These dropouts will be replaced.

Statistical Analysis:
Data will be presented as means ± SD or median (minimum; maximum) depending on normal distribution. Normal distribution will be checked by data visualization and the Kolmogorv- Smirnov test.
Due to the exploratory design of the study, there will be no correction for multiple testing. Due to the exploratory design of the study, there will be no correction for multiple testing.
Hepatic lipid content (HCL) will be considered as primary outcome parameter.
Nullhypothesis: There is no difference in HCL between patients with ACS and the control group.
Alternative hypothesis: There is a difference in HCL between patients with ACS and the control group.
Aim 1: comparisons between the groups of patients with ACS and the control group without ACS will be performed by unpaired student’s t-tests. In case of a large distribution of age between the groups, an adjustment by a regression model will be performed.
Aim 2: Comparisons of metric variables between the groups of patients will be performed by unpaired student’s t-tests or Mann- Whitney-U tests, depending on normal distribution. Chi-squared test will be used for comparison of discrete variables.
Aim 3: longitudinal analysis before and after treatment will be performed by using paired student’s t-tests.
Associations between continuous variables will be described by Pearson`s correlation coefficient.
SPSS - Statistics will be used for all computations. Level of statistical significance is set at $p<0.05$.

14. Present Personnel:

Dr. Peter Wolf (principal investigator, MD, expert in metabolic MRS studies): responsible for study design, coordination and supervision of the metabolic studies including data integration and analysis.

Dr. Michael Krebs (co-investigator, MD, Medical supervisor) responsible for study design, coordination and supervision of the metabolic studies including 1H-MRS, data integration and study design.

Dr. Michael Leutner (MD, experienced in metabolic studies): responsible for study design, patients' recruitment and coordination of individual experiments

Dr. Clemens Baumgartner (MD, PhD student): will be responsible for patients' recruitment and supervise the performance of individual experiments

Dr. Hannes Beiglböck (MD, experienced in metabolic studies): will be responsible for patients' recruitment and supervise the performance of individual experiments

Dr. Martin Krššák (National Collaborator, NMR-physicist, specialist in medical physics and biophysics) is an expert in the field of MRS, where he published many peer-reviewed articles. He will supervise the MRS measurements. His collaboration within the Division of Endocrinology is longstanding and our joint publishing effort will help him to further substantiate his scientific impact beyond NMR physics into the field of metabolism.

Dr. Helene Schernthaner-Reiter (consultant at the endocrinology outpatient's clinic): logistic support and patients' recruitment

Dr. Greisa Vila (Head of the Endocrinology outpatients' clinic): logistic support and expertise

Lakshmi Kalathinkunnath, MSc (Platform Rhythms of Life, University of Vienna): design and performance of the chronobiology analysis

Prof. Dr. Kristin Tessmar-Raible (Head of the Platform Rhythms of Life, University of Vienna): logistic support and expertise for the setup for the chronobiology analysis

Prof. Dr. Helmut Leder (Department of Cognition, Emotion, and Methods in Psychology): logistic support and expertise for the setup for the chronobiology analysis

o. Univ. Prof. Dr. Siegfried Trattnig, logistic support and expertise in MRI and MRS (Medical Scientific Head of Centre of Excellence, High Field MR, Medical University of Vienna)

o. Univ. Prof. Dr. Alexandra Kautzky-Willer (Head of the Department of Internal Medicine III, logistic support)

15. References:

1. Petramala L, Olmati F, Concistrè A, Russo R, Mezzadri M, Soldini M, Vincentis G De, Iannucci G, Toma G De, & Letizia C. Cardiovascular and metabolic risk factors in patients with subclinical Cushing. *Endocrine* 2020 . (doi:10.1007/s12020-020-02297-2)
2. Dalmazi G Di, Vicennati V, Garelli S, Casadio E, Rinaldi E, Giampalma E, Mosconi C, Golfieri R, Paccapelo A, Pagotto U, & Pasquali R. Cardiovascular events and mortality in patients with adrenal incidentalomas that are either non-secreting or associated with intermediate phenotype or subclinical Cushing's syndrome: a 15-year retrospective study. *The Lancet Diabetes and Endocrinology* 2014 **2** 396–405. (doi:10.1016/S2213-8587(13)70211-0)
3. Nieman LK, Biller BMK, Findling JW, Murad MH, Newell-Price J, Savage MO, & Tabarin A. Treatment of Cushing's Syndrome: An Endocrine Society Clinical Practice Guideline. *The Journal of Clinical Endocrinology & Metabolism* 2015 **100** 2807–2831. (doi:10.1210/jc.2015-1818)
4. Chiodini I. Diagnosis and treatment of subclinical hypercortisolism. *Journal of Clinical Endocrinology and Metabolism* 2011 **96** 1223–1236. (doi:10.1210/jc.2010-2722)
5. Fassnacht M, Arlt W, Bancos I, Dralle H, Newell-Price J, Sahdev A, Tabarin A, Terzolo M, Tsagarakis S, & Dekkers OM. Management of adrenal incidentalomas: European Society of Endocrinology Clinical Practice Guideline in collaboration with the European Network for the Study of Adrenal Tumors. *European journal of endocrinology* 2016 **175** G1–G34. (doi:10.1530/EJE-16-0467)
6. Lacroix A, Feelders RA, Stratakis CA, & Nieman LK. Cushing's syndrome. *The Lancet* 2015 **386** 913–927. (doi:10.1016/S0140-6736(14)61375-1)
7. Negri M, Pivonello C, Simeoli C, Gennaro G Di, Venneri MA, Sciarra F, Ferrigno R, Angelis C de, Sbardella E, Martino MC De, Colao A, Isidori AM, & Pivonello R. Cortisol Circadian Rhythm and Insulin Resistance in Muscle: Effect of Dosing and Timing of Hydrocortisone Exposure on Insulin Sensitivity in Synchronized Muscle Cells. *Neuroendocrinology* 2020 . (doi:10.1159/000512685)
8. Morris CJ, Purvis TE, Hu K, & Scheer FAJL. Circadian misalignment increases cardiovascular disease risk factors in humans. *Proceedings of the National Academy of Sciences of the United States of America* 2016 **113** E1402–E1411. (doi:10.1073/pnas.1516953113)
9. Pivonello R, Leo M De, Cozzolino A, & Colao A. The treatment of cushing's disease. *Endocrine Reviews* 2015 **36** 385–486. (doi:10.1210/er.2013-1048)
10. Debono M, Harrison RF, Chadarevian R, Gueroult C, Abitbol JL, & Newell-Price J. Resetting the abnormal circadian cortisol rhythm in adrenal incidentaloma patients with mild autonomous cortisol secretion. *Journal of Clinical Endocrinology and Metabolism* 2017 **102** 3461–3469. (doi:10.1210/jc.2017-00823)
11. Chanson P & Salenave S. Metabolic syndrome in Cushing's syndrome. *Neuroendocrinology* 2010 **92** 96–101. (doi:10.1159/000314272)
12. Wolf P, Winhofer Y, Krššák M, & Krebs M. Heart, lipids and hormones. *Endocrine Connections* 2017 **6** R59–R69. (doi:10.1530/EC-17-0031)
13. Kamenický P, Redheuil A, Roux C, Salenave S, Kachenoura N, Raissouni Z, Macron L, Guignat L, Jublanc C, Azarine A, Brailly S, Young J, Mousseaux E, & Chanson P. Cardiac structure and function in cushing's syndrome: A cardiac magnetic resonance imaging study. *Journal of Clinical Endocrinology and*

Metabolism 2014 **99** E2144–E2153. (doi:10.1210/jc.2014-1783)
14. Maurice F, Gaborit B, Vincentelli C, Abdesselam I, Bernard M, Graillon T, Kober F, Brue T, Castinetti F, & Dutour A. Cushing Syndrome Is Associated With Subclinical LV Dysfunction and Increased Epicardial Adipose Tissue. *Journal of the American College of Cardiology* 2018 **72** 2276–2277. (doi:10.1016/j.jacc.2018.07.096)
15. Wolf P, Marty B, Bouazizi B, Kachenoura N, Piedvache C, Blanchard A, Salenave S, Prigent M, Jublanc C, Ajzenberg C, Droumaguet C, Young J, Lecoq A, Kuhn E, Agostini H, Trabado S, Carlier P, Fève B, Redheuil A, Chanson P, & Kamenický P. Epicardial and pericardial adiposity without myocardial steatosis in Cushing's syndrome. *The Journal of Clinical Endocrinology & Metabolism* 2021 .
16. Pas R Van Der, Leebeek FWG, Hofland LJ, Herder WW De, & Feelders RA. Hypercoagulability in Cushing's syndrome: Prevalence, pathogenesis and treatment. *Clinical Endocrinology* 2013 **78** 481–488. (doi:10.1111/cen.12094)
17. Scherholz ML, Schlesinger N, & Androulakis IP. Chronopharmacology of glucocorticoids. *Advanced Drug Delivery Reviews* 2019 **151**–**152** 245–261. (doi:10.1016/j.addr.2019.02.004)
18. Wolf P, Mayr J, Beiglböck H, Fellinger P, Winhofer Y, Poglitsch M, Gessl A, Kautzky-willer A, Luger A, & Krebs M. Identifying a disease-specific renin-angiotensin-aldosterone system fingerprint in patients with primary adrenal insufficiency. *European Journal of Endocrinology* 2019 **181** 39–44.
19. O'Neill JS & Reddy AB. Circadian Clocks in Human Red Blood Cells. *Nature* 2011 **469** 498–503. (doi:10.1038/nature09702.Circadian)
20. Venneri MA, Hasenmajer V, Fiore D, Sbardella E, Pofi R, Graziadio C, Gianfrilli D, Pivonello C, Negri M, Naro F, Grossman AB, Lenzi A, Pivonello R, & Isidori AM. Circadian rhythm of glucocorticoid administration entrains clock genes in immune cells: A DREAM trial ancillary study. *Journal of Clinical Endocrinology and Metabolism* 2018 **103** 1–15. (doi:10.1210/jc.2018-00346)
21. Wolf P, Krššák M, Winhofer Y, Anderwald CH, Zwettler E, Kukurová IJ, Gessl A, Trattnig S, Luger A, Baumgartner-Parzer S, & Krebs M. Cardiometabolic phenotyping of patients with familial hypocalcuric hypercalcemia. *Journal of Clinical Endocrinology and Metabolism* 2014 **99** E1721–E1726. (doi:10.1210/jc.2014-1541)
22. Roenneberg T, Kuehnle T, Juda M, Kantermann T, Allebrandt K, Gordijn M, & Merrow M. Epidemiology of the human circadian clock. *Sleep medicine reviews* 2007 **11** 429–438. (doi:10.1016/j.smrv.2007.07.005)
23. Schmidt C, Collette F, Cajochen C, & Peigneux P. A time to think: circadian rhythms in human cognition. *Cognitive neuropsychology* 2007 **24** 755–789. (doi:10.1080/02643290701754158)
24. White D, Guilbert D, Varela VPL, Jenkins R, & Burton AM. GFMT2: A psychometric measure of face matching ability. *Behavior research methods* 2022 **54** 252–260. (doi:10.3758/s13428-021-01638-x)
25. Eddy CM & Hansen PC. Predictors of performance on the Reading the Mind in the Eyes Test. *PloS one* 2020 **15** e0235529. (doi:10.1371/journal.pone.0235529)
26. Greenwald AG, Nosek BA, & Banaji MR. Understanding and using the implicit association test: I. An improved scoring algorithm. *Journal of personality and social psychology* 2003 **85** 197–216. (doi:10.1037/0022-3514.85.2.197)
27. Mitrovic A, Hegelmaier LM, Leder H, & Pelowski M. Does beauty capture the eye, even if it's not (overtly) adaptive? A comparative eye-tracking study of

spontaneous attention and visual preference with VAST abstract art. *Acta psychologica* 2020 **209** 103133. (doi:10.1016/j.actpsy.2020.103133)

28. Lim U, Monroe K, Buchthal S, Fan B, Cheng I, Kristal B, Lampe J, Hullar M, Franke A, Stram D, Wilkens L, Shepherd J, Ernst T, & Marchand L Le. Propensity for Intra-abdominal and Hepatic Adiposity Varies Among Ethnic Groups. *Gastroenterology* 2019 **156** 966–975. (doi:10.1053/j.gastro.2018.11.021.Propensity)
